# High-Throughput Sequencing and the Viromic Study of Grapevine Leaves: From the Detection of Grapevine-Infecting Viruses to the Description of a New Environmental *Tymovirales* Member

**DOI:** 10.3389/fmicb.2018.01782

**Published:** 2018-08-29

**Authors:** Jean-Michel Hily, Thierry Candresse, Shahinez Garcia, Emmanuelle Vigne, Mélanie Tannière, Véronique Komar, Guillaume Barnabé, Antoine Alliaume, Sophie Gilg, Gérard Hommay, Monique Beuve, Armelle Marais, Olivier Lemaire

**Affiliations:** ^1^UMR 1131 Santé de la Vigne et Qualité du Vin, INRA-Université de Strasbourg, Colmar, France; ^2^UMR 1332 Biologie du Fruit et Pathologie, INRA, Univ. Bordeaux, Villenave d'Ornon, Bordeaux, France

**Keywords:** grapevine, high-throughput sequencing, virome, new virus, tymovirales, taxonomy

## Abstract

In the past decade, high-throughput sequencing (HTS) has had a major impact on virus diversity studies as well as on diagnosis, providing an unbiased and more comprehensive view of the virome of a wide range of organisms. Rather than the serological and molecular-based methods, with their more “reductionist” view focusing on one or a few known agents, HTS-based approaches are able to give a “holistic snapshot” of the complex phytobiome of a sample of interest. In grapevine for example, HTS is powerful enough to allow for the assembly of complete genomes of the various viral species or variants infecting a sample of known or novel virus species. In the present study, a total RNAseq-based approach was used to determine the full genome sequences of various grapevine fanleaf virus (GFLV) isolates and to analyze the eventual presence of other viral agents. From four RNAseq datasets, a few complete grapevine-infecting virus and viroid genomes were *de-novo* assembled: (a) three GFLV genomes, 11 grapevine rupestris stem-pitting associated virus (GRSPaV) and six viroids. In addition, a novel viral genome was detected in all four datasets, consisting of a single-stranded, positive-sense RNA molecule of 6033 nucleotides. This genome displays an organization similar to *Tymoviridae* family members in the *Tymovirales* order. Nonetheless, the new virus shows enough differences to be considered as a new species defining a new genus. Detection of this new agent in the original grapevines proved very erratic and was only consistent at the end of the growing season. This virus was never detected in the spring period, raising the possibility that it might not be a grapevine-infecting virus, but rather a virus infecting a grapevine-associated organism that may be transiently present on grapevine samples at some periods of the year. Indeed, the *Tymoviridae* family comprises isometric viruses infecting a wide range of hosts in different kingdoms (Plantae, Fungi, and Animalia). The present work highlights the fact that even though HTS technologies produce invaluable data for the description of the sanitary status of a plant, in-depth biological studies are necessary before assigning a new virus to a particular host in such metagenomic approaches.

## Introduction

Grapevine is one of the oldest domesticated crops and has been cultivated for more than seven millennia in a wide range of geographical areas (McGovern, 2003). To-date, over 70 viruses and five viroids have been identified as infecting grapevine (Martelli, 2017), making it the crop affected by the largest number of viral agents so far. While most plant viruses have probably co-existed with their hosts before domestication, others likely represent novel pathogen-host interactions. Many grapevine-infecting viruses or viroids have been detected in all grapevine-growing region within the last decade (Al Rwahnih et al., 2009, 2012, 2016; Navarro et al., 2009; Coetzee et al., 2010; Zhang et al., 2011, 2014; Giampetruzzi et al., 2012; Poojari et al., 2013; Beuve et al., 2015; Jo et al., 2017a,b; Silva et al., 2017; Blouin et al., 2018a,b; Candresse et al., 2018; Diaz-Lara et al., 2018), which is probably due to a combination of many factors such as: (i) the vegetative multiplication and international trade, (ii) newer and wider areas of cultivation associated with additional and different viral reservoir pool leading to potential spill-over (Perry et al., 2016), (iii) climate change with latent virus being awaken (Jones, 2015), (iv) a greater number of research being completed on such a high-profit/valuable crop, and (v) the use of the newest deep-sequencing technology (HTS, high-throughput sequencing) serving as a very sensitive diagnostic tool (Adams et al., 2009; Candresse et al., 2014).

In the last decade with the advent of HTS technologies, many microorganisms and their complex interactions within an ecosystem have been minutely described (Poinar et al., 2006; Kristensen et al., 2010; Qin et al., 2010; Suen et al., 2010). These new insights on the complex connection between microbial communities and their hosts contributed to a better description of the tree of life (Hug et al., 2016), but also to the elaboration of new concepts (Roossinck, 2011; Vayssier-Taussat et al., 2014; Cadwell, 2015) and the remodeling of old theories into new ones. In the pathology field, the most prominent advance would likely be the adaptation of Koch's original postulates, morphing from the simplistic “1 pathogen = 1 disease” equation to considering microbial interactions and their adaptation dynamics in order for the host to develop a disease, sometimes referred to as the “pathobiome” concept (Stecher et al., 2012; Byrd and Segre, 2016). However, after this first descriptive step, more work is needed in the field of etiology and functional genomics, in order to better understand the interactions between the microbiota, the pathogens and the host that might trigger the expression of disease and to precisely understand to which agents the pathobiome concept is most relevant.

In plant virology, a wide range of HTS approaches have been developed, leading to the detection of well-known viruses but also to the discovery of a wide range of novel plant viruses, greatly enriching our vision of the “virome” or “epigenome,” which refers to the exhaustive collection of nucleic acids that constitute the viral community *sensu-lato* associated with a particular host or ecosystem. In order to take into account viral diversity, many extraction methods have been perfected, focusing on the viral genetic make-up at different stages of the viral cycle: (i) total RNA or total DNA, with or without specific enrichment steps (Dayaram et al., 2012; Beuve et al., 2018), (ii) double-stranded RNA which targets viruses with dsRNA genomes as well as RNA viruses and viroids during their replication step (Coetzee et al., 2010; Blouin et al., 2016; Beuve et al., 2018), (iii) vsiRNA (viral small-interfering RNA) derived from the adaptive antiviral plant defense mechanism (Donaire et al., 2009; Kreuze et al., 2009), and (iv) encapsidated nucleic acid using the VANA (Virion-Associated Nucleic Acid) approach (Filloux et al., 2015). While each method may have some drawbacks (e.g., highly purified, DNAse-treated dsRNA not efficient for the detection of DNA viruses, non-encapsidated virus, or viruses with unstable particles missed by VANA), the large panel of HTS approaches available provides the investigator or diagnostician with the opportunity to fine-tune technical options to meet his specific objectives. HTS approaches have also been used at the ecosystem level in ecogenomic and metageogenomic studies (Roossinck et al., 2010; Bernardo et al., 2017). These large-scale studies assess the spatial and temporal distribution of plant virus populations within specific ecosystems, helping deciphering key components of viral evolution and disease emergence that shape wild and cultivated habitats in important agro-ecological interfaces (Alexander et al., 2014). In addition, some of the HTS-based approaches allow for full genome assembly, facilitating genome-wide studies (De Souza et al., 2017; Hily et al., 2018a; Muller et al., 2018), which were rarely feasible during the Sanger-sequencing era. Finally, RNA seq-based techniques can provide comprehensive transcriptomic analyses enabling the evaluation of gene expression between different phenological stages or varieties (Zenoni et al., 2010; Massonnet et al., 2017), the monitoring responses to environmental constraints (such as drought, temperature) (Haider et al., 2017; Londo et al., 2018) or the study of plant responses to specific infectious agent (Gambino et al., 2012; Blanco-Ulate et al., 2015). The counterpart is also possible, with the study of the impact of the culture of a particular genotype onto its natural environment (e.g., genetically modified organism risk assessment) (Hily et al., 2018b).

The *Tymovirales* order has been first established in 2004 and confirmed to regroup five families the *Alpha*-, *Beta*-, *Delta*-, *Gammaflexiviridae*, and the *Tymoviridae* families in 2009 (https://talk.ictvonline.org/taxonomy/p/taxonomy-history?taxnode_id=20172171, last visited 05/2018). Many viruses infecting grapevine have been either confirmed or identified via HTS techniques, with at least five of which [grapevine asteroid mosaic-associated virus (GAMaV), grapevine rupestris vein feathering virus (GRVFV), grapevine Syrah virus-1 (GSyV-1), grapevine fleck virus (GFkV), and grapevine red-globe virus (GRGV)] are part of the *Tymoviridae* family. While *Tymoviridae*, similar to the *Tymovirales* order in general, is predominantly a plant-infecting virus family (Edwards et al., 1997; Martelli et al., 2002; Elbeaino et al., 2011; Agindotan et al., 2012; Dutta et al., 2014), several of its members come from a wider range of hosts, such as insects (Wang et al., 2012; de Miranda et al., 2015) and, more recently, from the pathogenic fungus *Fusarium graminearum* (Li et al., 2016), the causal agent of Fusarium head blight of cereals. The *Tymoviridae* family consists of around 30 assigned virus species separated in three genera, *Tymovirus, Marafivirus*, and *Maculavirus*, while about 20 more virus species remained unassigned within the family/order (Table S1). Members of this family have many common characteristics (Martelli et al., 2002; King et al., 2012), such as: (i) non-enveloped isometric virions of about 30 nm (only family within the *Tymovirales* with such spherical particle, while the rest of this viral order is being composed of “flexivirus”), (ii) a mono-partite, positive, single-stranded RNA genome (6.0–7.5 kb in length), (iii) a generally high cytosine content (32–50% range), and (iv) a genomic RNA with a 5′ cap and a 3′-end with either a tRNA-like structure (*Tymoviruses*) or a polyA stretch (*Marafiviruses* and *Maculaviruses*). Other than members of the genus *Maculavirus* and the unassigned mycovirus Fusarium graminearum mycotymovirus 1 (FgMTV1) (Li et al., 2016), all other *Tymoviridae* present a highly conserved 16-nt sequence located at the end of the large coding sequence for the replication-associated polyprotein. Known as the “tymobox” or the “marafibox” (Ding et al., 1990; Izadpanah et al., 2002), it is believed to be an important element for the expression of a subgenomic messenger RNA for subsequent translation.

Here, using an RNAseq approach, our goal was to better characterize the sanitary status of Gewurztraminer scions grafted onto Kober 5BB rootstocks mono-infected with specific grapevine fanleaf virus (GFLV) isolates. Other than the near ubiquitous grapevine rupestris stem pitting-associated virus (GRSPaV) and viroids, we confirmed the presence of GFLV in the inoculated vines. Surprisingly, a thorough analysis of the RNASeq datasets revealed the presence of a new virus. Phylogenetic analyses showed this new virus to cluster within the *Tymovirales* order and to displays some but not all of the hallmarks of the *Tymoviridae* family. The sequence of this virus present in the grapevine environment is divergent enough to consider it as a new species typifying a new genus. The new virus is tentatively named grapevine-associated tymo-like virus (GaTLV) and the new genus tentatively named *Gratylivirus* (GRApevine TYmo-LIke).

## Materials and methods

### Plant material and conditions

Grapevine material used in this study came from a virus' *core*-collection maintained in an open-field by the Institut National de la Recherche Agronomique (INRA) in its Colmar research center (48.064457 lat., 7.334899 long.) (Table S2). Virus sources were isolated from GFLV-infected grapevine and biologically cloned via multiple passages on herbaceous hosts (Legin et al., 1993; Vigne et al., 2005, 2015; Hemmer et al., 2018; Hily et al., 2018a). Viral isolates were inoculated to Kober 5BB (*Vitis berlandieri* X *V. riparia*, clone 259) rootstocks using a heterologous grafting technique. Finally, certified virus-free *Vitis vinifera* cv. Gewurztraminer (clone 643 from INRA Colmar collection) were grafted onto healthy and GFLV-infected rootstock for further study (Vigne et al., 2015).

*Nicotiana benthamiana* [wild-type, NbDCLx mutant (Andika et al., 2015) and transgenic B2 (Monsion et al., 2018)] and *Chenopodium quinoa* were grown in growth chambers kept at 22/18°C (day/night) with a 14/10 h light/dark photoperiod. Similar settings were also used for the mechanical transmission experiments.

For the epidemiological study, all details about the samples used are presented in Table 1. Samples were flash frozen in liquid nitrogen and kept at −80°C prior to analysis.

**Table 1:** Biological samples used in this study.

**Section**	**Sample name**	**Cultivar**	**Sampling location**	**Origin**	**Technique**	**Sampling date**	**GaTLV**	**GAPDH**
1st observation	EVC53	Gewurztraminer	Alsace-Colmar-INRA-open field	Colmar, Fr	Illumina 2^*^150	05/09/2013	✓	Na
	EVC42	Gewurztraminer	Alsace-Colmar-INRA-open field	Colmar, Fr	Illumina 2^*^150	05/09/2013	✓	Na
	EVC60	Gewurztraminer	Alsace-Colmar-INRA-open field	Colmar, Fr	Illumina 2^*^150	05/09/2013	✓	Na
	EVC56	Gewurztraminer	Alsace-Colmar-INRA-open field	Colmar, Fr	Illumina 2^*^150	05/09/2013	✓	Na
Epidemiology linked to timing	*V. sylvestris*	Na	Alsace-Colmar-INRA-open field	Grau du roi, Fr	RT-PCR	18/05/2011	✗	NT
	*V. rupestris*	Na	Alsace-Colmar-INRA-open field	Nd	RT-PCR	17/06/2008	✗	NT
	*V. candicans*	Na	Alsace-Colmar-INRA-open field	Nd	RT-PCR	17/06/2008	✗	NT
	*V. ishikari*	Na	Alsace-Colmar-INRA-open field	Nd	RT-PCR	17/06/2008	✗	NT
	*V. davidii*	Na	Alsace-Colmar-INRA-open field	Nd	RT-PCR	17/06/2008	✗	NT
	*V. amurensis*	Na	Alsace-Colmar-INRA-open field	Nd	RT-PCR	17/06/2008	✗	NT
	*A. japonica*	Na	Alsace-Colmar-INRA-open field	Nd	RT-PCR	17/06/2008	✗	NT
	*A. cordata*	Na	Alsace-Colmar-INRA-open field	Nd	RT-PCR	17/06/2008	✗	NT
	*A. peunculata*	Na	Alsace-Colmar-INRA-open field	Nd	RT-PCR	17/06/2008	✗	NT
	Y276	Otscha Bala	Alsace-Colmar-INRA-open field	Ouzbekistan	RT-PCR	06/08/2012	✗	NT
	95-184	Grand Noir	Alsace-Colmar-INRA-open field	Volos, Greece	RT-PCR	11/09/2014	✓	NT
	P119	Zirock	Alsace-Colmar-INRA-open field	Nd	RT-PCR	12/09/2014	✓	NT
	C1200	Chardonnay	Alsace-Colmar-INRA-open field	Nd	RT-PCR	22/09/2014	✓	NT
	N37	Cabernet franc	Alsace-Colmar-INRA-open field	Nd	RT-PCR	22/09/2014	✓	NT
	96-29	Lambrusco Lambo	Alsace-Colmar-INRA-open field	Italy	RT-PCR	26/09/2012	✓	NT
	Z170	Ruby Cabernet	Alsace-Colmar-INRA-open field	France	RT-PCR	26/09/2012	✓	NT
	96-60	Nebbiolo	Alsace-Colmar-INRA-open field	Italy	RT-PCR	26/09/2012	✓	NT
	Y245	Karasakis	Alsace-Colmar-INRA-open field	Turquie	RT-PCR	27/09/2012	✓	NT
	96-42	Nebbiolo	Alsace-Colmar-INRA-open field	Italy	RT-PCR	02/10/2012	✓	NT
	Y259	Lutea	Alsace-Colmar-INRA-open field	Italy	RT-PCR	02/10/2012	✓	NT
	Y270	Muscat d′Istambul	Alsace-Colmar-INRA-open field	Israël	RT-PCR	02/10/2012	✓	NT
	E4	Redglobe	Alsace-Colmar-INRA-open field	IFV-ENTAV	RT-PCR	24/10/2012	✓	NT
	Y206	Chaouch rose	Alsace-Colmar-INRA-open field	Turkey	RT-PCR	25/10/2011	✗	NT
	BB2-6	Touriga Francesa	Alsace-Colmar-INRA-open field	Portugal	RT-PCR	25/10/2011	✗	NT
	95-151	Alphonse Lavallée	Alsace-Colmar-INRA-open field	IFV-ENTAV	RT-PCR	29/10/2012	✓	NT
	T113	Chatus	Alsace-Colmar-INRA-open field	Bordelais	RT-PCR	09/11/2011	✓	NT
Timing confirmation	EVC53	Gewurztraminer	Alsace-Colmar-INRA-open field	Colmar, Fr	RT-PCR	20/06/2016	✗	NT
	EVC53	Gewurztraminer	Alsace-Colmar-INRA-open field	Colmar, Fr	RT-PCR	11/07/2016	✗	✓
	EVC53	Gewurztraminer	Alsace-Colmar-INRA-open field	Colmar, Fr	RT-PCR	29/08/2016	✗	NT
	EVC53	Gewurztraminer	Alsace-Colmar-INRA-open field	Colmar, Fr	RT-PCR	22/09/2016	✓	✓
	EVC60	Gewurztraminer	Alsace-Colmar-INRA-open field	Colmar, Fr	RT-PCR	20/06/2016	✗	NT
	EVC60	Gewurztraminer	Alsace-Colmar-INRA-open field	Colmar, Fr	RT-PCR	11/07/2016	✗	✓
	EVC60	Gewurztraminer	Alsace-Colmar-INRA-open field	Colmar, Fr	RT-PCR	29/08/2016	✗	NT
	EVC60	Gewurztraminer	Alsace-Colmar-INRA-open field	Colmar, Fr	RT-PCR	22/09/2016	✓	✓
	Tu1	Nd	Alsace-Turckheim-Abandonned	Nd	RT-PCR	30/05/2017	✗	NT
	Tu2	Nd	Alsace-Turckheim-Abandonned	Nd	RT-PCR	30/06/2017	✗	NT
	Tu3	Nd	Alsace-Turckheim-Abandonned	Nd	RT-PCR	30/07/2017	✓	NT
	Win1	Nd	Alsace-Wintzenheim-ResDur	Nd	RT-PCR	30/05/2017	✗	NT
	Win2	Nd	Alsace-Wintzenheim-ResDur	Nd	RT-PCR	30/06/2017	✗	NT
	Win3	Nd	Alsace-Wintzenheim-ResDur	Nd	RT-PCR	30/07/2017	✓	NT
Open field vs. Greenhouse	EVC49	Gewurztraminer	Alsace-Colmar-INRA-open field	Colmar, Fr	RT-PCR	22/09/2016	✓	✓
	EVA4	Gewurztraminer	Alsace-Colmar-INRA-open field	Colmar, Fr	RT-PCR	22/09/2016	✓	✓
	EVA13	Gewurztraminer	Alsace-Colmar-INRA-open field	Colmar, Fr	RT-PCR	22/09/2016	✓	✓
	EVA16	Gewurztraminer	Alsace-Colmar-INRA-open field	Colmar, Fr	RT-PCR	22/09/2016	✓	✓
	EVA20	Gewurztraminer	Alsace-Colmar-INRA-open field	Colmar, Fr	RT-PCR	22/09/2016	✓	✓
	EVA24	Gewurztraminer	Alsace-Colmar-INRA-open field	Colmar, Fr	RT-PCR	22/09/2016	✓	✓
	EVA49	Gewurztraminer	Alsace-Colmar-INRA-open field	Colmar, Fr	RT-PCR	22/09/2016	✓	✓
	A	Nd	Alsace-Colmar-INRA-open field	Nd	RT-PCR	27/09/2016	✓	✓
	B	Nd	Alsace-Colmar-INRA-open field	Nd	RT-PCR	27/09/2016	✓	✓
	C	Nd	Alsace-Colmar-INRA-open field	Nd	RT-PCR	27/09/2016	✓	✓
	D	Nd	Alsace-Colmar-INRA-open field	Nd	RT-PCR	27/09/2016	✓	✓
	Kober 5BB healthy	Na	Alsace-Colmar-INRA-Greenhouse	Grau du roi, Fr	RT-PCR	27/09/2016	✓	✓
	Kober 5BB GFLVinf1	Na	Alsace-Colmar-INRA-Greenhouse	Grau du roi, Fr	RT-PCR	27/09/2016	✗	✓
	Kober 5BB GFLVinf2	Na	Alsace-Colmar-INRA-Greenhouse	Grau du roi, Fr	RT-PCR	27/09/2016	✗	✓
	Kober 5BB GFLVinf3	Na	Alsace-Colmar-INRA-Greenhouse	Grau du roi, Fr	RT-PCR	27/09/2016	✓	✓
	Gw cl643	Gewurztraminer	Alsace-Colmar-INRA-Greenhouse	Grau du roi, Fr	RT-PCR	27/09/2016	✗	✓
	Chard cl131	Chardonnay	Alsace-Colmar-INRA-Greenhouse	Grau du roi, Fr	RT-PCR	27/09/2016	✗	✓
	ENTAV-E39	11R-cl237	Alsace-Colmar-INRA-Greenhouse	Grau du roi, Fr	RT-PCR	27/09/2016	✗	✓
	ENTAV-E173	Grenache-cl435	Alsace-Colmar-INRA-Greenhouse	Grau du roi, Fr	RT-PCR	27/09/2016	✗	✓
	ENTAV Kober 5BB cl114	Na	Alsace-Colmar-INRA-Greenhouse	Grau du roi, Fr	RT-PCR	27/09/2016	✗	✓
	Cramant N332	Chardonnay	Alsace-Colmar-INRA-Greenhouse	Cramant, Fr	RT-PCR	27/09/2016	✗	✓
	Bennwhir BE/5-47 N79	Gewurztraminer	Alsace-Colmar-INRA-Greenhouse	Bennwhir, Fr	RT-PCR	27/09/2016	✗	✓
	Chablis 39-1 N24	Chardonnay	Alsace-Colmar-INRA-Greenhouse	Chablis, Fr	RT-PCR	27/09/2016	✗	✓
Epidemiology	E	Nd	Alsace-Eguisheim	Nd	RT-PCR	27/09/2016	✓	✓
	F	Nd	Alsace-Husseren les Chateaux	Nd	RT-PCR	27/09/2016	✓	✓
	G	Nd	Alsace-Voegtlinshoffen	Nd	RT-PCR	27/09/2016	✓	✓
	H	Nd	Alsace-Gueberschwihr	Nd	RT-PCR	27/09/2016	✓	✓
	I	Nd	Alsace-Grand cru Hatschbourg	Nd	RT-PCR	27/09/2016	✓	✓
	J	Nd	Alsace-Herrlisheim	Nd	RT-PCR	27/09/2016	✓	✓
	Mes	Nd	Cognac-Messac	Nd	RT-PCR	01/11/2016	✗	✓
	Ger	Nd	Cognac-Germignac	Nd	RT-PCR	01/11/2016	✗	✓
	Sal	Nd	Cognac-Salignac	Nd	RT-PCR	01/11/2016	✗	✓
	TT2017-73	Nd	Burgundy-Villers la Faye	Nd	Illumina 2^*^150	11/09/2017	✓	Na
	TT2017-74	Nd	Burgundy-Villers la Faye	Nd	Illumina 2^*^150	11/09/2017	✓	Na
	TT2017-75	Nd	Burgundy-Villers la Faye	Nd	Illumina 2^*^150	11/09/2017	✓	Na
	TT2017-76	Nd	Burgundy-Villers la Faye	Nd	Illumina 2^*^150	11/09/2017	✓	Na
	TT2017-77	Nd	Burgundy-Villers la Faye	Nd	Illumina 2^*^150	11/09/2017	✓	Na
	TT2017-78	Nd	Burgundy-Villers la Faye	Nd	Illumina 2^*^150	11/09/2017	✗	Na
	TT2017-79	Nd	Burgundy-Villers la Faye	Nd	Illumina 2^*^150	11/09/2017	✓	Na
	TT2017-80	Nd	Burgundy-Villers la Faye	Nd	Illumina 2^*^150	11/09/2017	✓	Na
Superficial	Swab EVC53	Na	Alsace-Colmar-INRA-open field	Colmar, Fr	RT-PCR	27/09/2016	✓	✗
	Swab EVC60	Na	Alsace-Colmar-INRA-open field	Colmar, Fr	RT-PCR	27/09/2016	✗	✓
	Swab EVC56	Na	Alsace-Colmar-INRA-open field	Colmar, Fr	RT-PCR	27/09/2016	✓	✓
	Negative Swab	Na	Na	Na	RT-PCR	27/09/2016	✓	✓
Fungi	*Alternaria tenuissima*	Gewurztraminer	Alsace-Colmar-INRA-open field	Na	RT-PCR	27/09/2016	✗	Na
	*Leptosphaerulina chartarum*	Gewurztraminer	Alsace-Colmar-INRA-open field	Na	RT-PCR	27/09/2016	✗	Na
	*Epicoccum nigrum*	Gewurztraminer	Alsace-Colmar-INRA-open field	Na	RT-PCR	27/09/2016	✗	Na
	*Aureobasidium pullulans*	Gewurztraminer	Alsace-Colmar-INRA-open field	Na	RT-PCR	27/09/2016	✗	Na
	*Cladosporium cladosporioides*	Gewurztraminer	Alsace-Colmar-INRA-open field	Na	RT-PCR	27/09/2016	✗	Na
	*Dothioraceae sp*	Gewurztraminer	Alsace-Colmar-INRA-open field	Na	RT-PCR	27/09/2016	✗	Na
	*Penicillium oxalicum*	Gewurztraminer	Alsace-Colmar-INRA-open field	Na	RT-PCR	27/09/2016	✗	Na
	*Cladosporium* sp.	Gewurztraminer	Alsace-Colmar-INRA-open field	Na	RT-PCR	27/09/2016	✗	Na
	*Fusarium equiseti*	Gewurztraminer	Alsace-Colmar-INRA-open field	Na	RT-PCR	27/09/2016	✗	Na
	*Cryptococcus magnus*	Gewurztraminer	Alsace-Colmar-INRA-open field	Na	RT-PCR	27/09/2016	✗	Na
	*Botrytis cinerea*	Gewurztraminer	Alsace-Colmar-INRA-open field	Na	RT-PCR	27/09/2016	✗	Na
	*Mucor circinelloides*	Gewurztraminer	Alsace-Colmar-INRA-open field	Na	RT-PCR	27/09/2016	✗	Na
	*Plasmopara viticola*	Chardonnay	Alsace-Colmar-INRA-open field	Na	RT-PCR	2006	✗	Na
	*Erysiphe necator*	Na	Alsace-Colmar-INRA-open field	Na	RT-PCR	2016	✗	Na
	*Guignardia bidwellii*	Na	Alsace-Colmar-INRA-open field	Na	RT-PCR	2015	✗	Na

*Relevant information relative to samples used in this study are shown, comprising: section (describing the purpose of sample use), names, cultivars, and sampling location, origin of the sample, techniques used to detect GaTLV, sampling dates. Na, Not applicable; Nd, Not determined; NT, Not tested; ✓: detected, ✗: absence*.

### Fungi isolation and cultivation

Leaves from field-grown grapevine plants that tested positive for the new virus in September 2017 were dipped into Nanopure^TM^ water (Thermo Fisher Scientific, Waltham, MA, USA) and slowly shaken for 15 min. A few microliters of the solution or of a 1/100th dilution were spread onto three different selective media, Peptone Yeast Extract Agar, Potato Dextrose Agar, and Dichloran Rose Bengal Agar (all from Sigma-Aldrich, St Louis, MO, USA) and the plates incubated at room temperature for a few days. Fungal mycelia were isolated and maintained for further studies such as testing for the presence of the new virus or fungal ITS barcoding (White et al., 1990; Gardes and Bruns, 1993). *Plasmopara viticola* (downy mildew), *Erysiphe necator* (powdery mildew), and *Guignardia bidwellii* (black rot) isolated from the same open-field trial and maintained in laboratory conditions were kindly provided by Sabine Wiedemann-Merdinoglu and were similarly tested. Briefly, mycelia were recovered and ground with mortar and pestle after addition of Fontainebleau sand (MERCK eurolab, Briare le canal, FR) and 400 μL of Nanopure^TM^ water. The mixture was then placed at 95°C for 15 min and then kept at −80°C prior to RNA and DNA extraction (see below).

### Mechanical transmission attempts

Attempts to propagate the new virus by mechanical inoculation were carried out using two grapevine infected leaf tissue samples (EVC53 and EVC60). Freshly collected leaves were ground at a 1:5 ratio [wt:vol] in 5 ml of a modified Sorensen's phosphate buffer (35 mM Na2HPO4, 15 mM KH2PO4, pH 7.2) without or supplemented with 2.5% nicotine. In each case, 20 plants of *C. quinoa*, wild type *Nicotiana benthamiana*, as well as transgenic B2 plants, and *N. benthamiana NbDCLx*, that are affected in their natural silencing defense mechanism and known to greatly promote virus multiplication, were tested.

### RNA and DNA extraction, cDNA amplification, and high throughput sequencing

Total RNAs were extracted from 100 mg of leaf tissue, or 100 μl of mycelial homogenate, using the RNeasy Plant mini kit (Qiagen, Venlo, Netherlands), as per manufacturer's recommendations. Post extraction, for cDNA library preparation and Illumina sequencing, purity criteria (A260/A230 and A260/A280 both >1.8) and quality levels (RIN>8) were assessed via Nanodrop™ (Thermo Fisher Scientific Inc., Waltham, MA, USA) and Bioanalyzer (Agilent, Santa Clara, CA, USA). The cDNA libraries were then prepared and processed at IGBMC “microarray and sequencing platform” facility (Strasbourg, France). Paired-end 2 × 150 pb RNAseq was performed on a Hiseq 2500 (Illumina, San Diego, CA, USA) following the manufacturer's instructions.

For fungal DNA extraction, a CTAB-based extraction method was used (Saghai-Maroof et al., 1984).

### HTS data analyses

Analyses of dataset were performed using the CLC Genomics Workbench 8.5.1 software (CLC bio Genomics, Aarhus, Denmark). After the trimming and quality check procedure, only reads above 70 nucleotides (nts) were kept (see Table S3).

The sanitary status of the grapevine samples was assessed by mapping reads onto a curated collection of grapevine viruses reference (Martelli, 2017) as previously described (Hily et al., 2018b). A relaxed mapping stringency (0.5 read length/0.7 similarity) was used in order to take into account genome diversity within each virus species. RPKM values, expressing the abundance of viral reads in each sample were calculated taking into account the number of reads mapping to each reference virus, its length, and the total number of reads from the sample. In parallel, *de novo* assembly was performed after removal of reads that mapped onto the *Vitis vinifera* genome (http://www.plantgdb.org/XGDB/phplib/download.php?GDB=Vv, Genoscope 12x, last visited 05/2018). Contigs were then tested against GenBank reference sequences using BlastN/BlastX (http://blast.ncbi.nlm.nih.gov/Blast.cgi, last visited 05/2018).

### *In silico* sequence analysis and statistical analyses

Nucleic acid and deduced amino acid products were analyzed using CLC Genomics Workbench 8.5.1. The GaTLV nucleotide and deduced protein sequences were compared with other viral sequences from GenBank and EMBL databases using the FASTA (Pearson and Lipman, 1988) and BLAST (Altschul et al., 1990) programs. Identity and similarity percentages were obtained using mean length of sequences, BLOSUM62 matrix with gap cost of 10 and gap extension of 0.5. Alignment analysis and tentative Maximum Likelihood-based phylogenetic trees of amino acid sequences were performed using the MUSCLE (Edgar, 2004) and MEGA7 (Kumar et al., 2016) softwares. The best ML-fitted model for each sequence alignments was used and bootstrapping analyses of 100 replicates were performed. Trees were visualized using iTOL (Letunic and Bork, 2016).

Protein structure, modeling, and structural similarity match to Protein Data Bank (PDB) were performed using the I-TASSER suite (Yang et al., 2015) (http://zhanglab.ccmb.med.umich.edu/I-TASSER/, last visited 05/2018). Correlation coefficients were determined using the statistical software package Statgraphics Centurion version 15.1.02 (StatPoint technologies, Inc., Warrenton, VA, USA).

### Molecular analyses

A 3′/5′ RACE kit (SMARTer RACE, Clontech Lab., CA, USA) was used for the amplification and confirmation of the viral genome termini, as per manufacturer's recommendations. The virus-specific primers (including a 15 bp overlap sequence for cloning purposes in italic), GSP fwd (5′-*GATTACG*CCAAGCTTGTCAACGGGTTATTTGATGGCGGAGGGTG) and GSP rev (5′-*GATTACGC*CAAGCTTCGCGGTACCAAACGTTCACGCTCACC) were used along with oligo UPM (Clontech Lab.) to amplify the genome 3′ and 5′ ends, respectively. Resulting PCR products were Sanger-sequenced, confirming already known sequences and allowing termini to be resolved.

Amplification of the putative CP coding sequence was performed using primers containing attB1 and attB2 sequences (in italic) upstream and downstream of the coding region (with start and stop codon underlined): fwd 5′-*GGGGACAAGTTTGTACAAAAAAGCAGGCTTCATG*TCTGAGATTACACCCGTGC and rev 5′-*GGGGACCACTTTGTACAAGAAAGCTGGGTCTCA*AGCAAAAACAATATCGTAACCAT. PCR products were then cloned by Gateway® recombination, following the manufacturer's protocol (Invitrogen, Carlsbad, USA), into successively pDONR/Zeo donor vector and pEAQ- *HT*-Dest1 binary plasmid (Peyret and Lomonossoff, 2013). The resulting plasmid was then used for attempting VLP production (Belval et al., 2016). A plasmid containing GFP was used as positive control. The same primers, but without the attB sequences, were used for viral detection.

For fungal identification, a barcoding analysis based on the internal transcribed sequences (ITS) which consisted in amplifying a genomic region with primers ITS1F (CTTGGTCATTTAGAGGAAGTAA) and ITF4 (TCCTCCGCTTATTGATATGC) followed by Sanger sequencing.

## Results

### Analysis of the full sanitary status of GFLV-infected plants and initial characterization of a novel virus

RNASeq datasets obtained from a collection of Gewurztraminer grapevines singly infected by GFLV isolates were first analyzed by directly mapping total cleaned reads (Table S3) onto a curated collection of grapevine viruses' reference sequences. Of the four plants tested, the EVC53 grapevine was used as a negative control in our laboratory, as it was not inoculated with GFLV. Its sanitary status was confirmed by HTS (Table 2), since no viruses nor viroids were detected, other than the three near ubiquitous agents of grapevine; grapevine rupestris stem pitting-associated virus (GRSPaV), hop stunt viroid (HSVd) and grapevine yellow-speckle viroid 1 (GYSVd1). On the other hand, in addition to GRSPaV, HSVd and GYSVd1, all three other tested grapevines samples (EVC42, EVC56, and EVC60) displayed, as expected, reads mapping on GFLV RNA1, and RNA2 sequences. EVC56 was the only sample to exhibit reads corresponding to the satellite RNA3 sequence (Table 2). No other grapevine-infecting viruses/viroids were detected in any of the tested plants using this “direct-mapping” approach (data not shown).

**Table 2:** Sanitary status.

			**GFLV consensus**				**Viroids**				
			**RNA1**	**RNA2**	**RNA3**	**GRSPaV**	**HSVd**	**GYSVd1**	**GaTLV**	**Contigs related to**	
**Sample name**	**Total clean reads**	**Size (nt)**	**7,341**	**3,750**	**1,114**	**8,725**	**298**	**366**	**6,033**	**Insect**	**Fungi**
**EVC53**	13,147,378	**reads #**	**22**	**50**	**0**	**7,853**	**564**	**396**	**627**		
healthy grapevine		*RPKM*	*0*	*1*	*0*	*68*	*144*	*82*	*8*	*10*	*144*
		Genome	0	0	0	2	2	2	1		
**EVC42**	12,258,388	**reads #**	**77,887**	**92,619**	**13**	**6,341**	**1,214**	**747**	**3,247**		
GFLV-infected		*RPKM*	*701*	*1,630*	*1*	*48*	*269*	*135*	*44*	*7*	*174*
		Genome	1	2	0	3	2	1	1		
**EVC60**	14,210,779	**reads #**	**48,433**	**53,089**	**15**	**9,209**	**1,098**	**504**	**12,588**		
GFLV-infected		*RPKM*	*464*	*996*	*1*	*74*	*259*	*97*	*147*	*18*	*659*
		Genome	1	1	0	4	1	1	1		
**EVC56**	16,543,942	**reads #**	**52,660**	**61,265**	**33,687**	**4,202**	**1,048**	**500**	**1,547**		
GFLV-infected		*RPKM*	*434*	*988*	*1,828*	*29*	*213*	*83*	*15*	*19*	*264*
		Genome	1	1	1	2	2	2	1		

*Number of reads (in bold) and RPKM (Reads Per Kilobase per Million reads mapped to the reference, in italic) for each grapevine viruses and viroids found in the four samples analyzed. Genome: correspond to the number of complete (to near complete) genomes assembled in de novo. GFLV, grapevine fanleaf virus; GRSPaV, grapevine rupestris stem pitting-associated virus; GaTLV, grapevine-associated tymo-like virus; HSVd, Hop stunt viroid, and GYSVd1, grapevine yellow speckle viroid-1*.

*Reads were mapped on a set of reference viruses previously described as infecting Vitis vinifera. For mapping, a low stringency was used with parameters set to 0.5 for read length and 0.7 for similarity. Size of the sequences tested is noted*.

To confirm this initial analysis, a *de novo* assembly was performed, allowing for contigs and scaffolds reconstruction. In this way, near-complete genomes for each of the viruses/viroids infecting all four samples tested, consisting in three new GFLV-RNA1 sequences, four GFLV-RNA2, one GFLV-RNA3, 11 GRSPaV as well as four HSVd, and three GYSVd1 viroid sequences were obtained (Table 2, Table S2). For each samples, after removal of sequences corresponding to known viruses and viroids, the remaining contigs were annotated using BlastN/BlastX. Most contigs corresponded to grapevine transcriptome and were set aside. A few remaining contigs from the different samples displaying high percentage of identity among each other were of interest, with the largest of them displaying a maximum length of 4,900 nt. Using BlastN, this contig did not reveal any significant homology with any other GenBank sequence. However, at the amino acid level and using BlastX, the predicted encoded protein revealed homologies with members of the *Tymoviridae* family, with the presence of conserved domains of a viral methyltransferase (MTR), a peptidase (PRO), a helicase (Hel), and a polymerase (RdRp) with very strong e-values (1 e^−8^ or lower) but with only low amino acid sequence identity levels, close to 30%. The sequence was further manually extended by several rounds of read mapping, until no more reads mapped against it. This allowed to obtain a continuous contig of 6,030 nt (including a stretch of seven adenines ending the sequence at its 3′ end). To confirm terminal sequences, 3′- and 5′-RACE reactions were performed. The complete genome sequence of the new virus was thus established to be 6,033 nt (without the polyA tail), indicating that only nine bases at the 5′-end had been missing from the original completed assembly. For all tested samples, final read counts mapping onto this new complete virus genome are shown in Table 2. The virus was detected in all tested samples, but with a very large variation in representation, with average sequencing depth varying between 14.6X (EVC53) and 291X (EVC60) (Table 2). No other viral contigs were identified in any of the tested samples.

### Genome organization and phylogenetic association of the new virus with the *Tymovirales* order

**Figure 1:**
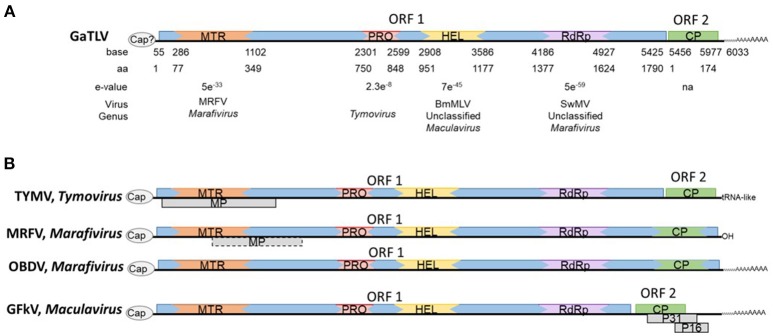
**(A)** Genetic organization of grapevine-associated tymo-like virus, GaTLV. Domain's signatures, location (bases and amino-acids) and BlastP e-values with corresponding virus and genus according to each domains are shown below. MTR, Methyltransferase; MP, Movement protein; PRO, Peptidase-C21; HEL, viral Helicase, and RdRp, Polymerase of the RdRp2 superfamily. **(B)** Schematic representation of different genome organization of various members of the family *Tymoviridae;* TYMV, *Turnip yellow mosaic virus*; MRFV, *Maize rayado fino virus*; OBDV, *Oat blue dwarf virus*, and GFkV, *Grapevine fleck virus*.

Analyses of the new virus genome organization revealed two open reading frames (ORFs) (Figure 1A). The first ORF encodes a large replication-associated polypeptide of 1,790 amino acid (p203), which accounts for the majority of the coding capacity of the genome. Functional domains were detected using BlastP and are conserved with viruses of the alpha-like superfamily of positive-strand RNA viruses (locations and e-values are shown in Figure 1A). For example, the Hel domain (aa 951–1177) contains a sequence (^953^GYPGCGKT^960^) analogous to the Hel motif I [GxxGxGK(T/S)] of many viral NTP-binding proteins, well conserved within the *Tymovirales* order. The second ORF does not display any similarities with any GenBank sequences at the nucleic acid level nor at the amino acid level. Also no putative conserved domains could be detected in ORF2. More elements on this second ORF are detailed in the next section.

**Figure 2:**
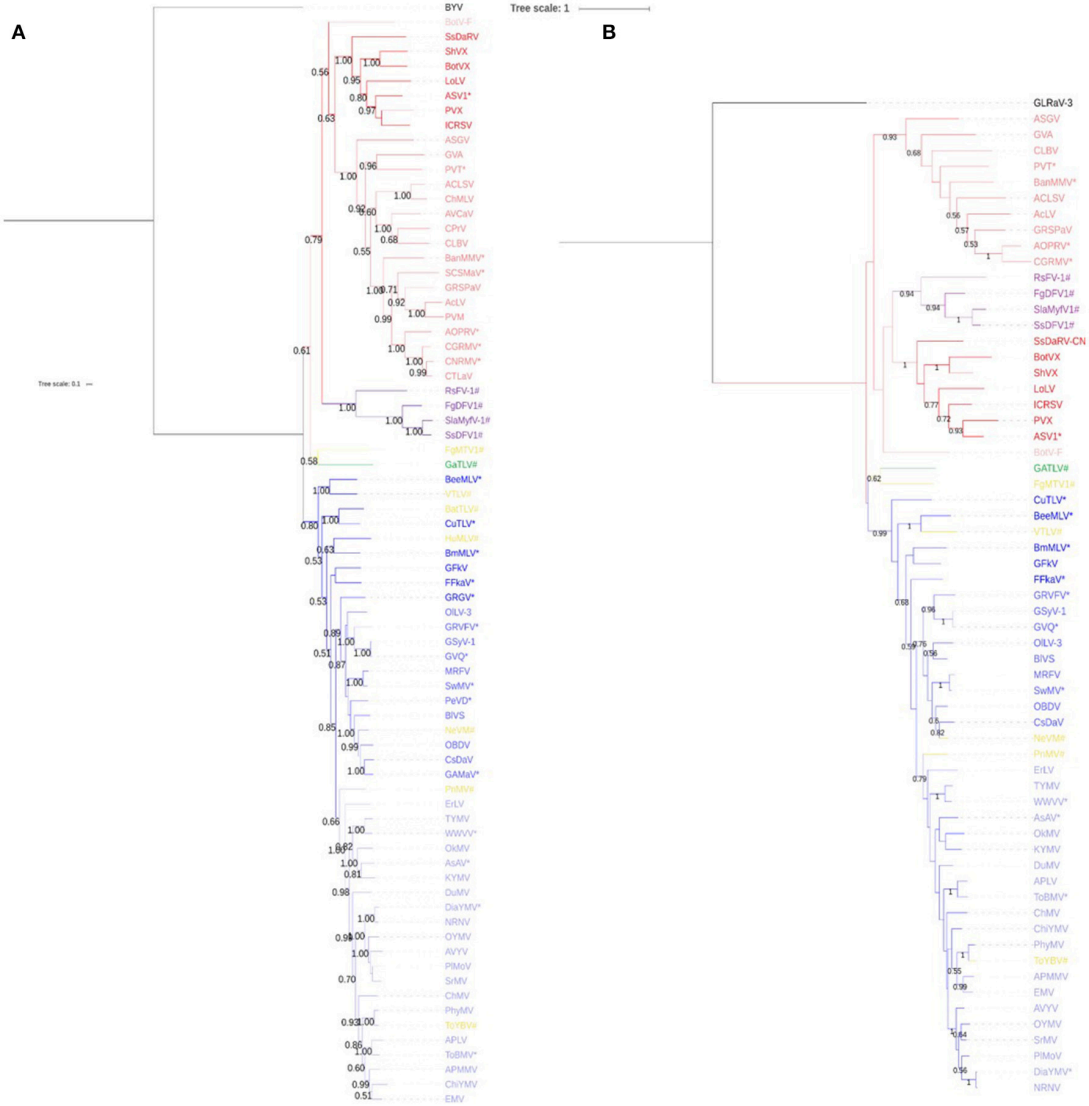
Phylogenetic trees based on the Maximum likelihood algorithm of **(A)** ORF1 amino acid sequence and **(B)** MTR protein domain of GaTLV (in green) compared to other viruses of the *Tymovirales* order with *Alphaflexiviridae* (Darker red), *Betaflexiviridae* (red), *Deltaflexiviridae* (purple), *Gammaflexiviridae* (light red), and *Tymoviridae* (different shade of blue), with maculaviruses (navy blue), marafiviruses (blue), and tymoviruses (light blue). Unassigned virus within the order are shown in yellow and ^#^. ^*^ indicates unclassified virus within a genus. Bootstrap values over 0.50 are shown.

Phylogenetic analyses were performed on a multiple alignment of the ORF1-encoded proteins of *Tymovirales* members (Figure 2A, Figure S1). While the new virus replication-associated protein (REP) clustered within the *Tymovirales* order, it could not be assigned to a particular family as it clustered away from members of the various families that constitute this order. Similar analyses performed using only the most conserved regions of the REP proteins (MTR, Hel, and RdRP, Figure 2B, Figures S2A,B, respectively) confirmed the affinities of the new virus with the *Tymovirales*, but again without providing a definitive message as to its affinities within this order. Although the closest associations tended to be with *Tymoviridae* members.

While the new viral sequence could not definitely be assigned to a particular family within the *Tymovirales* using the REP protein phylogenetic analyses, the virus shares many properties with the *Tymoviridae* family. (i) The genome organization and order of the conserved domains along the large ORF1 are most consistent with those of *Tymoviridae* members (Figure 1). (ii) As indicated above, closest affinities for all REP conserved motifs are with *Tymoviridae* family members (Figure 1A), with all genera (*Tymovirus, Marafivirus*, and *Maculavirus*) being identified and represented. For example, the RdRp motifs are well conserved, especially the REP motif IV (^1517^ANDYTSFDQSQTGE^1530^) and REP-VI (^1605^VSGDD^1609^), with that of other members of the *Tymoviridae*. This was confirmed by looking at amino acid identity levels of all conserved domains, with the *Tymoviridae* percentages being generally higher than with any other *Tymovirales* (Table S4). (iii) The genome size is in the low range but typical of the family (6.0–7.5 kp) (Martelli et al., 2002). (iv) The absence of the AlkB conserved domain or of a Triple Gene Block (TGB) or 30K-like movement protein module is also typical of the *Tymoviridae* as opposed to the other families comprised in the *Tymovirales*.

However, some typical features of the *Tymoviridae* family are not observed in the new virus. For example, the “Tymobox” or “marafibox,” a very distinctive 16 nt region thought to control subgenomic RNA synthesis and present in most *Tymoviridae* (except for two members, GFkV, and FgMTV1), was not identified in the genome of the new virus. In addition, unlike for other *Tymoviridae*, for which an unusually high cytosine (C) content (32–50%) is observed, the new virus sequence exhibited a strong unbalanced content in C but in the opposite direction, with only 15.2 % (Adenine count: 27.7%, Tymine: 32.2%, and Guanine: 24.9%).

### Characterization of the putative capsid protein

While no sequence similarities were found using the BLASTN/X/P tools, the secondary structure as well as residues accessibility of the protein encoded by this second ORF were predicted. *In silico* modeling of this protein was performed via the I-TASSER suit website and models were then compared to the Protein Data Bank (PDB) library (data not shown). While partial sequence identity were fairly low (<18%), the best model hits corresponded to the capsid proteins of a few *Tymoviruses* [e.g., desmodium yellow mottle tymovirus (DYMV), turnip mosaic virus (TuMV), and physalis mottle virus (PhMV)] (Krishna et al., 1999; Larson et al., 2000, 2005) and a *Sobemovirus* (sesbania mosaic virus, SeMV) (Bhuvaneshwari et al., 1995). The TM-alignment program was then used to match proteins at the structure level in order to pinpoint and further identify a potential ORF2 protein function. All nine PDB hits corresponded to viral capsid proteins, with the three best hits corresponding to *Tymoviridae*'s CPs (TM-scores of 0.826, 0.821, and 0.817, for DYMV, TuMV, and PhMV capsid protein element structures, respectively, Figure S3) and the last one to the hepatitis E virus (HEV) (TM-score of 0.712).

To try to validate ORF2 as the coding sequence for the capsid protein, the complete ORF2 sequence (from start to stop codon included) was cloned in a pEAQ-based expression vector in order to produce *in planta* the protein in the hope to generate VLPs as previously described (Belval et al., 2016). Despite the fact that the plasmid sequence was conform by Sanger sequencing and that a GFP-positive plasmid control expressed the fluorescent protein, no VLPs could be observed (data not shown).

### Epidemiology and potential host of the new virus

To better decipher the origin and the biological significance of the new virus, mechanical inoculation of crude leaf extracts were undertaken. None of the 80 plants tested (consisting of 20 plants of either wild-type *N. benthamiana* or *C. quinoa*, as well as *N. benthamiana* B2, and NbDCLx, both accessions highly susceptible to virus and known to greatly promote virus multiplication) displayed any symptoms nor tested positive for the virus by RT-PCR.

To further study the distribution, the prevalence and the diversity of the virus, more than 70 samples were screened for its presence via RT-PCR. All leaf samples used in this study are presented in Table 1. First, we tested 26 samples from the grapevine “*core*-collection” maintained in an experimental plot at INRA-Colmar. Those samples were chosen since they covered a wide range of grapevine varieties, various phenological growth stages and different sampling dates and years. Surprisingly, more than half of the samples tested positive for the presence of the virus (Table 1, “epidemiology linked to timing” section). Interestingly, all positive samples seemed to have been collected at the end of the growing season (September or later). RT-PCR-based amplicons were then Sanger sequenced. Over a span of 404 nts within ORF2, a maximum of only six single nucleotide polymorphisms (SNPs) was detected, showing a high level of sequence conservation (>98.5%) between isolates. Testing of further samples from different origins, the seasonal detection of the new virus was confirmed and detected as early as the mid-summer from either abandoned vineyards (Turckheim, Alsace, Fr) or lightly treated plots (Wintzenheim, Alsace, Fr) but only from September on for the rest of the samples (Table 1, “timing confirmation” and “open field vs. greenhouse” sections). Remarkably, from samples collected in September, the detection rate was higher in samples collected from open-field (100%), than in samples maintained under greenhouse conditions (8%). All aforementioned results seemed to imply a relatively loose association of the virus with grapevine, possibly reflecting an “environmental” component to the presence of the virus in the tested grapevine samples. So far, the virus was found to be present in areas surrounding the INRA Colmar research station, and, more broadly, along the “Route des vins d'Alsace” (Table 1, “epidemiology” section). However, the virus was not detected in a few samples collected from the Cognac region. In addition, we never found any reads corresponding to its sequence in any of our other grapevine HTS datasets, corresponding to about 120 samples that span areas such as the Alsace, Champagne and Chablis regions (data not shown). Nonetheless, it should be considered that most of these samples had been collected in the spring/early summer when the titer grapevine fanleaf virus is at its peak. Interestingly, the new virus was however identified recently, in the frame of a study supported by the BIVB (Interprofessional Office of Burgundy Wines), in seven out of eight samples collected in early Autumn in four plots of Pinot Noir and Chardonnay in Burgundy, France (Table 1, “epidemiology” section). Six complete (to near complete) genome sequences could be assembled from these samples and showed a maximum of 22 mutations (six of them coding) along the complete genome as compared to the Colmar isolate reference sequence (EVC60), corresponding to a minimum of 99.6% nucleotide identity. These results confirm both the temporal association of this new virus with grapevine and its low level of diversity.

This loose association with grapevine was reinforced by experiments in which the surface of grapevine leaves was swept gently with a cotton swab from which total RNAs were then extracted and tested. While plant RNAs were not detected in this way (all samples tested negative in a RT-PCR assay for grapevine glyceraldehyde 3-phosphate dehydrogenase gene), the virus was detected in two out of the tree tested samples (Table 1, “superficial” section), suggesting that the virus might rather be a surface contaminant of grapevine leaves rather than a grapevine-infecting virus.

Since some *Tymovirales*, and more specifically *Tymoviridae* members, are known to infect fungi or insects, an attempt was made at correlating the titer of the new virus with the presence of contigs annotated as coming from insects or fungi in the original RNASeq datasets (Table 2). A correlation was found between the presence of the virus and insects-derived contigs but was not statistically supported (CC = 0.43; *R*^2^ = 0.18, *p*-value = 0.571). Conversely, a statistically valid and very robust positive correlation was observed between the new viral sequence and the presence of contigs identified as originating from fungi (CC = 0.96, *R*^2^ = 0.92, *p*-value = 0.039), strongly suggesting that this new virus could in fact be a novel mycovirus. Following this hypothesis, a total of 15 fungal species were isolated from grapevine leaves and berries at a time when the virus was detected (Table 1, “fungi” section). While all fungal isolates were readily identified using an ITS barcoding technique, none tested positive for the presence of the new virus by RT-PCR. During the same period, a few insect species were trapped and tested for the presence of the virus. As for fungi, our effort to attribute an insect host to this new virus was fruitless (data not shown).

## Discussion

With the dawn of HTS and the huge amounts of data being produced from a single experiment, many field of research have been significantly impacted. With the rise of HTS technology emerged new concepts, old theories, and postulates being modified and remodeled (e.g., Koch's original postulate) (Byrd and Segre, 2016). From medicine, clinical diagnostic, microbiology to ecology, plant pathology (and more specifically plant virology) seems to have benefited the most from these new tools. Grapevine research also improved with HTS, with many new viruses being identified all over the world, belonging to different families such as *Betaflexiviridae* (Al Rwahnih et al., 2012; Giampetruzzi et al., 2012; Jo et al., 2017a,b; Blouin et al., 2018a,b; Candresse et al., 2018; Diaz-Lara et al., 2018), *Caulimoviridae* (Zhang et al., 2011), *Luteoviridae* (Silva et al., 2017), *Secoviridae* (Al Rwahnih et al., 2016), or *Tymoviridae* (Al Rwahnih et al., 2009; Beuve et al., 2015; Cretazzo and Velasco, 2017; Vargas-Asencio et al., 2017). HTS technologies have been confirmed to be a powerful diagnostic tool allowing for an exhaustive description of viral species present in many grapevine sample (Coetzee et al., 2010; Al Rwahnih et al., 2011; Jo et al., 2015; Beuve et al., 2018). Depending on the chosen methodology, complete (to near complete) viral genome can be also be assembled, re-shaping the viral evolution field (Simmonds et al., 2017) with genome-wide studies made easily achievable (Hily et al., 2018a).

In this present study, our initial goal to better define the sanitary status of grapevine plants, healthy or mono-infected with different GFLV isolates, was fulfilled since we obtained the complete genome sequences of the different GFLV isolates involved. All three RNA1, four RNA2, and one RNA3 complete sequences were submitted to GenBank (Table S2). This was performed using a dual strategy involving either direct mapping of reads against a collection of reference sequences of grapevine-infecting viruses and viroids or by *de novo* assembly of reads followed by BlastN/BlastX annotation of contigs. As expected, all samples displayed reads corresponding to ubiquitous grapevine viral pathogens: grapevine rupestris stem pitting-associated virus (GRSPaV) as well as two viroids (HSVd and GYSVd1) (Table 2). From each sample, two to four genomes of GRSPaV were assembled, confirming the fact that multiple GRSPaV variants can infect a single grapevine (Beuve et al., 2018). All 11 GRSPaV genomes thus obtained were part of a genome-wide diversity study of GRSPaV (Hily et al., 2018a). Altogether, this is another proof of the need to consider these viral or subviral agents as part of the grapevine “background” virome (Saldarelli et al., 2017). Surprisingly, the “healthy” grapevine (EVC53) displayed a few reads mapping onto GFLV references (Table 2). These reads were considered as a mild “intra-lane” contamination since all 72 reads covered less than 30% of the complete RNA1 plus RNA2 GFLV genome (e.g., ≈11 000 nt). Such contamination is often encountered in HTS datasets and is discussed in a method article in this same issue (Vigne et al., 2004). No other grapevine-infecting viruses were detected using the “direct-mapping” method.

Out of the thousands of contigs *de novo* assembled for each grapevine sample and after comparison against the NCBI database using BlastN/BlastX, some contigs showed a distant relationship (average aa identity close to 30%) to several viruses belonging to the *Tymoviridae* family and the *Marafivirus* genus in particular. Further steps ultimately yielded a genome of 6,033 nt (excuding the polyA tail). From this sequence, two ORFs were predicted. Phylogenetic analyses of the replicase-based polypeptide encoded by the ORF1, placed this new virus within the *Tymovirales* order, however no unambiguous assignment to a particular family could be attained as the new virus REP clustered away from members of the five families currently defined in the *Tymovirales*. Although not biologically confirmed, ORF2 was computationally described as coding for the coat protein after modeling and comparison to the Protein Data Bank (PDB) library. The viral coat proteins to which distant homologies were identified in this way all have icoseadral particles, suggesting the new virus could also have paraspherical particles, similar to members of the *Tymoviridae* family (Figure S3). This element could be added to the list of features shared with this family and outlined above. Yet, the very low C content (only 15.2%) and the REP phylogeny set the new virus aside from the *Tymoviridae* while other features exclude it from the known genera within the family. For example, the presence of a 3′ poly(A) disqualifies it to be part of the *Tymovirus* genus (Dreher, 2004), while the genome organization sets it aside from the *Marafivirus* and *Maculavirus* genera.

Considering the originality of the features of this new agent, and the inability to unequivocally assign it at this time in any existing families in the *Tymovirales* order, the safest option seem (i) to tentatively name it ‘*gra*pevine-associated *ty*mo-*li*ke virus’ or GaTLV and (ii) to consider that it defines a new genus (provisionally named *Gratylivirus*) that will remain in the order but unassigned to a particular family for the time being. More viruses closely related to GaTLV need to be described before a decision can be made to decide whether the genus *Gratylivirus* should be included in the *Tymoviridae* or be included in a novel family within the *Tymovirales*. The sequence of GaTLV was deposited to GenBank under accession number MH383239.

Viruses belonging to the *Tymovirirales* order are known to infect many different species covering different Kingdoms. They are mostly found in Plantae, but lately many have been described infecting Fungi as well as the class of Insecta in the Animalia Kingdom (King et al., 2012; Li et al., 2016). To further characterize GaTLV and discover its putative host(s), we first tried to propagate the virus in herbaceous plants via mechanical inoculation, which was unfortunately not successful. Such failure to identify alternative herbaceous hosts does not rule out grapevine as the original host. Indeed, it has been previously reported that some *Tymoviridae* display a narrow host range (Dreher, 2004; Alabdullah et al., 2010), up to the extreme situation of having a single identified host [e.g. maize rayado fino virus (MRFV) restricted to corn (Nault et al., 1980) or GFkV to *Vitis* spp. (King et al., 2012)]. A study spanning a wide range of samples was performed by RT-PCR (Table 1). While half of the samples tested positive for the presence of GaTLV, the virus was detected only in samples collected at the end of the summer/early autumn season, which corresponds to a period when fungicide/pesticide treatments are generally discontinued. Such connection with fungicide/pesticide applications is further emphasized by the comparison of the detection rates for samples collected in early autumn from open-field (all positive for the virus) and from greenhouses where fungicide treatments were still in use (less than 10% positive). In addition, a swipe test demonstrated that the virus seems to be loosely present on the surface of grapevine leaves and/or berries. Taken together all these evidences suggest that GaTLV is likely a surface contaminant on grapevine leaves and might therefore rather be a virus infecting insects or fungi. This hypothesis of GaTLV to be a mycovirus is reinforced by the strong positive correlation, statistically supported, observed between the presence of GaTLV, and that of fungi-derived contigs. Unfortunately, in an attempt to identify the potential host, none of the 15 isolated fungi tested positive for GaTLV, including some major grapevine pathogenic species such as *Botrytis cinerea, Plasmopara viticola, Erysiphe necator*, or *Guignardia bidwellii*.

From positive samples, a genetic diversity study was performed. When comparing all complete genomes from two different locations (Alsace and Burgundy), this virus displayed a very high identity percentage (>99.6%) along the genome. While the sampling size might be too small to be certain, this lack of diversity could underscore either a slow-evolving virus, a virus infecting a new host which did not have the time to accumulate substantial divergence or a virus highly specialized to its host. While none of the aforementioned experiments were conclusive, directly identifying a host, only correlative results were accumulated, tentatively pinpointing GaTLV as a mycovirus belonging to the grapevine phytobiome. More experiments are needed in order to uncover the host of GatLV, such as graft experiments that, if negative, would additionally support the notion that GaTLV is not a grapevine-infecting virus or controlled fungicide treatment of grapevines that could lend support to the hypothesis that GaTLV may be a mycovirus.

This work highlights the fact that even though HTS technologies produce an invaluable sum of information describing the sanitary status of a plant, a careful etiological and epidemiological study is necessary before assigning a new virus to a host. Nonetheless, in this work and as it is often the case following HTS analysis, even after a careful scientific investigation, it is still not possible to designate without any doubt the host of an infectious entity. Our study also confirm that grapevine phytobiome is probably richer than anticipated, with the use of HTS allowing for the detection of not only grapevine pathogens but also grapevine associated-ecosystem (Al Rwahnih et al., 2011; Espach et al., 2012).

## Data availability

All sequences *de novo* assembled have been submitted to GenBank (Table S2). The raw data supporting the conclusions of this manuscript will be made available by the authors, without undue reservation, to any qualified researcher.

## Author contributions

J-MH, TC, EV, and OL designed the experiments. J-MH, TC, SGa, MT, VK, GB, AA, SGi, GH, MB, and AM performed the experiments. J-MH, TC, EV, and OL wrote the paper. All authors contributed to manuscript revision, read, and approved the submitted version.

### Conflict of interest statement

The authors declare that the research was conducted in the absence of any commercial or financial relationships that could be construed as a potential conflict of interest.

## References

[B1] Adams I. P. Glover R. H. Monger W. A. Mumford R. Jackeviciene E. Navalinskiene M.. (2009). Next-generation sequencing and metagenomic analysis: a universal diagnostic tool in plant virology. Mol. Plant Pathol. 10, 537–545. 10.1111/j.1364-3703.2009.00545.x 19523106 PMC6640393

[B2] Agindotan B. O. Gray M. E. Hammond R. W. Bradley C. A. (2012). Complete genome sequence of switchgrass mosaic virus, a member of a proposed new species in the genus Marafivirus. Arch. Virol. 157, 1825–1830. 10.1007/s00705-012-1354-3 22661377

[B3] Al Rwahnih M. Alabi O. J. Westrick N. M. Golino D. Rowhani A. (2016). Near-complete genome sequence of grapevine fabavirus, a novel putative member of the genus fabavirus. Genome Announc. 4:e00703#x02013;16. 10.1128/genomeA.00703-16 27445385 PMC4956458

[B4] Al Rwahnih M. Daubert S. Golino D. Rowhani A. (2009). Deep sequencing analysis of RNAs from a grapevine showing Syrah decline symptoms reveals a multiple virus infection that includes a novel virus. Virology 387, 395–401. 10.1016/j.virol.2009.02.028 19304303

[B5] Al Rwahnih M. Daubert S. Úrbez-Torres J. R. Cordero F. Rowhani A. (2011). Deep sequencing evidence from single grapevine plants reveals a virome dominated by mycoviruses. Arch. Virol. 156, 397–403. 10.1007/s00705-010-0869-8 21140178 PMC3044836

[B6] Al Rwahnih M. Sudarshana M. R. Uyemoto J. K. Rowhani A. (2012). Complete genome sequence of a novel vitivirus isolated from grapevine. J. Virol. 86:9545. 10.1128/JVI.01444-12 22879616 PMC3416117

[B7] Alabdullah A. Minafra A. Elbeaino T. Saponari M. Savino V. Martelli G. P. (2010). Complete nucleotide sequence and genome organization of Olive latent virus 3, a new putative member of the family Tymoviridae. Virus Res. 152, 10–18. 10.1016/j.virusres.2010.05.010 20561953

[B8] Alexander H. M. Mauck K. E. Whitfield A. E. Garrett K. A. Malmstrom C. M. (2014). Plant-virus interactions and the agro-ecological interface. Eur. J. Plant Pathol. 138, 529–547. 10.1007/s10658-013-0317-1

[B9] Altschul S. F. Gish W. Miller W. Myers E. W. Lipman D. J. (1990). Basic local alignment search tool. J. Mol. Biol. 215, 403–410. 10.1016/S0022-2836(05)80360-2 2231712

[B10] Andika I. B. Maruyama K. Sun L. Kondo H. Tamada T. Suzuki N. (2015). Differential contributions of plant Dicer-like proteins to antiviral defences against potato virus X in leaves and roots. Plant J. 81, 781–793. 10.1111/tpj.12770 25619543

[B11] Belval L. Hemmer C. Sauter C. Reinbold C. J. Fauny D. Ritzenthaler C. (2016). Display of whole proteins on inner and outer surfaces of Grapevine fanleaf virus-like particles. Plant Biotechnol. J. 14, 2288–2299. 10.1111/pbi.12582 27178344 PMC5103221

[B12] Bernardo P. Charles-Dominique T. Barakat M. Ortet P. Fernandez E. Filloux D.. (2017). Geometagenomics illuminates the impact of agriculture on the distribution and prevalence of plant viruses at the ecosystem scale. ISME J. 12:173. 10.1038/ismej.2017.155 29053145 PMC5739011

[B13] Beuve M. Candresse T. Tannières M. Lemaire O. (2015). First report of grapevine redglobe virus (GRGV) in grapevine in France. Plant Dis. 99, 422–422. 10.1094/PDIS-10-14-1009-PDN 30699720

[B14] Beuve M. Hily J. M. Alliaume A. Reinbold C. Le Maguet J. Candresse T.. (2018). A complex virome unveiled by deep sequencing analysis of RNAs from a French Pinot Noir grapevine exhibiting strong leafroll symptoms. Arch. Virol. [Epub ahead of print]. 10.1007/s00705-018-3949-9 30033497

[B15] Bhuvaneshwari M. Subramanya H. S. Gopinath K. Savithri H. S. Nayudu M. V. Murthy M. R. (1995). Structure of sesbania mosaic virus at 3 A resolution. Structure 3, 1021–1030. 8589997 10.1016/s0969-2126(01)00238-6

[B16] Blanco-Ulate B. K. Amrine C. H. Collins T. S. Rivero R. M. Vicente A. R. Cantu D. (2015). Developmental and metabolic plasticity of white-skinned grape berries in response to *Botrytis cinerea* during noble rot. Plant Physiol. 169, 2422–2443. 10.1104/pp.15.00852 26450706 PMC4677888

[B17] Blouin A. G. Chooi K. M. Warren B. Napier K. R. Barrero R. A. MacDiarmid R. M. (2018a). Grapevine virus I, a putative new vitivirus detected in co-infection with grapevine virus G in New Zealand. Arch. Virol. 163, 1371–1374. 10.1007/s00705-018-3738-5 29392493

[B18] Blouin A. G. Keenan S. Napier K. R. Barrero R. A. MacDiarmid R. M. (2018b). Identification of a novel vitivirus from grapevines in New Zealand. Arch. Virol. 163, 281–284. 10.1007/s00705-017-3581-0 29026999

[B19] Blouin A. G. Ross H. A. Hobson-Peters J. O'Brien C. A. Warren B. MacDiarmid R. (2016). A new virus discovered by immunocapture of double-stranded RNA, a rapid method for virus enrichment in metagenomic studies. Mol. Ecol. Resourc. 16, 1255–1263. 10.1111/1755-0998.12525 26990372

[B20] Byrd A. L. Segre J. A. (2016). Adapting Koch's postulates. Science 351, 224–226. 10.1126/science.aad6753 26816362

[B21] Cadwell K. (2015). The virome in host health and disease. Immunity 42, 805–813. 10.1016/j.immuni.2015.05.003 25992857 PMC4578625

[B22] Candresse T. Filloux D. Muhire B. Julian C. Galzi S. Fort G. Roumagnac P.. (2014). Appearances can be deceptive: revealing a hidden viral infection with deep sequencing in a plant quarantine context. PLoS ONE 9:e102945. 10.1371/journal.pone.0102945 25061967 PMC4111361

[B23] Candresse T. Theil S. Faure C. Marais A. (2018). Determination of the complete genomic sequence of grapevine virus H, a novel vitivirus infecting grapevine. Arch. Virol. 163, 277–280. 10.1007/s,00705-017-3587-7 29018968

[B24] Coetzee B. M. Freeborough J. Maree H. J. J. Celton M. D. Rees J. G. Burger J. T. (2010). Deep sequencing analysis of viruses infecting grapevines: virome of a vineyard. Virology 400, 157–163. 10.1016/j.virol.2010.01.023 20172578

[B25] Cretazzo E. Velasco L. (2017). High-throughput sequencing allowed the completion of the genome of grapevine Red Globe virus and revealed recurring co-infection with other tymoviruses in grapevine. Plant Pathol. 66, 1202–1213. 10.1111/ppa.12669

[B26] Dayaram A. Opong A. Jäschke A. Hadfield J. Baschiera M. R. Varsani A. (2012). Molecular characterisation of a novel cassava associated circular ssDNA virus. Virus Res. 166, 130–135. 10.1016/j.virusres.2012.03.009 22465471

[B27] de Miranda J. R. Cornman R. S. Evans J. D. Semberg E. Haddad N. Neumann P.. (2015). Genome characterization, prevalence and distribution of a macula-like virus from *Apis mellifera* and varroa destructor. Viruses 7, 3586–3602. 10.3390/v7072789 26154017 PMC4517118

[B28] De Souza J. Müller G. Perez W. Cuellar W. Kreuze J. (2017). Complete sequence and variability of a new subgroup B nepovirus infecting potato in central Peru. Arch. Virol. 162, 885–889. 10.1007/s00705-016-3147-6 27858290 PMC5329089

[B29] Diaz-Lara A. Golino D. Al Rwahnih M. (2018). Genomic characterization of grapevine virus J, a novel virus identified in grapevine. Arch. Virol. 163, 1965–1967. 10.1007/s00705-018-3793-y 29516247 PMC5999178

[B30] Ding S. W. Howe J. Keese P. Mackenzie A. Meek D. Osorio-Keese M.. (1990). The tymobox, a sequence shared by most tymoviruses: its use in molecular studies of tymoviruses. Nucleic Acids Res. 18, 1181–1187. 10.1093/nar/18.5.1181 2320413 PMC330433

[B31] Donaire L. Wang Y. Gonzalez-Ibeas D. Mayer K. F. Aranda M. A. Llave C. (2009). Deep-sequencing of plant viral small RNAs reveals effective and widespread targeting of viral genomes. Virology 392, 203–214. 10.1016/j.virol.2009.07.005 19665162

[B32] Dreher T. W. (2004). Turnip yellow mosaic virus: transfer RNA mimicry, chloroplasts and a C-rich genome. Mol. Plant Pathol. 5, 367–375. 10.1111/j.1364-3703.2004.00236.x 20565613

[B33] Dutta M. Sokhandan Bashir N. Palmer M. W. Melcher U. (2014). Genomic characterization of Ambrosia asymptomatic virus 1 and evidence of other Tymovirales members in the Oklahoma tallgrass prairie revealed by sequence analysis. Arch. Virol. 159, 1755–1764. 10.1007/s00705-014-1985-7 24519459

[B34] Edgar R. C. (2004). MUSCLE: multiple sequence alignment with high accuracy and high throughput. Nucleic Acids Res. 32, 1792–1797. 10.1093/nar/gkh340 15034147 PMC390337

[B35] Edwards M. C. Zhang Z. Weiland J. J. (1997). Oat blue dwarf marafivirus resembles the tymoviruses in sequence, genome organization, and expression strategy. Virology 232, 217–229. 10.1006/viro.1997.8555 9185605

[B36] Elbeaino T. Digiaro M. Martelli G. P. (2011). Complete sequence of Fig fleck-associated virus, a novel member of the family Tymoviridae. Virus Res. 161, 198–202. 10.1016/j.virusres.2011.07.022 21840352

[B37] Espach Y. Maree H. J. Burger J. T. (2012). The use of next-generation sequencing to identify novel mycoviruses in single grapevine plants, in 17th International Council for the Study of Virus and Virus-like Diseases of the Grapevine (ICVG) (Davis, CA).

[B38] Filloux D. Dallot S. Delaunay A. Galzi S. Jacquot E. Roumagnac P. (2015). Metagenomics approaches based on virion-associated nucleic acids (VANA): an innovative tool for assessing without a priori viral diversity of plants, in Plant Pathology. Methods in Molecular Biology, ed Lacomme C. (New York, NY: Humana Press). 10.1007/978-1-4939-2620-6_18 25981259

[B39] Gambino G. Cuozzo D. Fasoli M. Pagliarani C. Vitali M. Boccacci P.. (2012). Co-evolution between Grapevine rupestris stem pitting-associated virus and Vitis vinifera L. leads to decreased defence responses and increased transcription of genes related to photosynthesis. J. Exp. Bot. 63, 5919–5933. 10.1093/jxb/ers,244 22987838

[B40] Gardes M. Bruns T. D. (1993). ITS primers with enhanced specificity for basidiomycetes - application to the identification of mycorrhizae and rusts. Mol. Ecol. 2, 113–118. 10.1111/j.1365-294X.1993.tb00005.x 8180733

[B41] Giampetruzzi A. Roumi V. Roberto R. Malossini U. Yoshikawa N. La Notte P.. (2012). A new grapevine virus discovered by deep sequencing of virus- and viroid-derived small RNAs in cv Pinot gris. Virus Res. 163, 262–268. 10.1016/j.virusres.2011.10.010 22036729

[B42] Haider M. S. Zhang C. Kurjogi M. M. Pervaiz T. Zheng T. Zhang C.. (2017). Insights into grapevine defense response against drought as revealed by biochemical, physiological and RNA-Seq analysis. Sci. Rep. 7:13134. 10.1038/s41598-017-13464-3 29030640 PMC5640638

[B43] Hemmer C. Djennane S. Ackerer L. Hleibieh K. Marmonier A. Gersch S.. (2018). Nanobody-mediated resistance to Grapevine fanleaf virus in plants. Plant Biotechnol. J. 16, 660–671. 10.1111/pbi.12819 28796912 PMC5787842

[B44] Hily J.- M. Demanèche S. Poulicard N. Tannières M. Djennane S.. Lemaire O. (2018b). Metagenomic-based impact study of transgenic grapevine rootstock on its associated virome and soil bacteriome. Plant Biotechnol. J. 16, 208–220. 10.1111/pbi.12761 28544449 PMC5785345

[B45] Hily J. M. Beuve M. Vigne E. Demangeat G. Candresse T. Lemaire O. (2018a). A genome-wide study of grapevine rupestris stem pitting-associated virus. Arch. Virol. [Epub ahead of print]. 10.1007/s00705-018-3945-0 30043203

[B46] Hug L. A. Baker B. J. Anantharaman K. Brown C. T. Probst A. J. Castelle C. J.. (2016). A new view of the tree of life. Nat. Microbiol. 1:16048. 10.1038/nmicrobiol.2016.48 27572647

[B47] Izadpanah K. Ping Zhang Y. Daubert S. Masumi M. Rowhani A. (2002). Sequence of the coat protein gene of bermuda grass etched-line virus, and of the adjacent ‘marafibox’ motif. Virus Genes 24 131–134. 10.1023/A:1014516515454 12018703

[B48] Jo Y. Choi H. Kyong Cho J. J. Yoon Y. S. Choi K. Kyong Cho W. (2015). In silico approach to reveal viral populations in grapevine cultivar Tannat using transcriptome. Data 5:15841. 10.1038/srep15841 26508692 PMC4623741

[B49] Jo Y. M. Song K. Choi H. J. Park S. J. Lee W. Cho W. K.. (2017b). Genome sequence of grapevine virus T, a novel foveavirus infecting grapevine. Genome Announc. 5:e00995–17. 10.1128/genomeA.00995-17 28912330 PMC5597771

[B50] Jo Y. M. Song K. Choi H. J. Park S. J. Lee W. Cho W. K.. (2017a). Genome sequence of grapevine virus K, a novel vitivirus infecting grapevine. Genome Announc. 5:e00994–17. 10.1128/genomeA.00994-17 28912329 PMC5597770

[B51] Jones G. V. (2015). Grapevines in a Changing Environment, Grapevine in a Changing Environment. New York, NY: John Wiley & Sons, Ltd 1–17.

[B52] King A. M. Q. Adams M. J. Cartens E. B. Lefkowitz E. J. (2012). Family - Tymoviridae, Virus Taxonomy, Ninth report of the International Committee on Taxonomy of Viruses. San Diego, CA: Elsevier Academic Press 944–952.

[B53] Kreuze J. F. Perez A. Untiveros M. Quispe D. Fuentes S. Barker I.. (2009). Complete viral genome sequence and discovery of novel viruses by deep sequencing of small RNAs: a generic method for diagnosis, discovery and sequencing of viruses. Virology 388, 1–7. 10.1016/j.virol.2009.03.024 19394993

[B54] Krishna S. S. Hiremath C. N. Munshi S. K. Prahadeeswaran D. Sastri M. Savithri H. S.. (1999). Three-dimensional structure of physalis mottle virus: implications for the viral assembly. J. Mol. Biol. 289, 919–934. 10.1006/jmbi.1999.2787 10369772

[B55] Kristensen D. M. Mushegian A. R. Dolja V. V. Koonin E. V. (2010). New dimensions of the virus world discovered through metagenomics. Trends Microbiol. 18, 11–19. 10.1016/j.tim.2009.11.003 19942437 PMC3293453

[B56] Kumar S. Stecher G. Tamura K. (2016). MEGA7: molecular evolutionary genetics analysis version 7.0 for bigger datasets. Mol. Biol. Evol. 33, 1870–1874. 10.1093/molbev/msw054 27004904 PMC8210823

[B57] Larson S. B. Day J. Canady M. A. Greenwood A. McPherson A. (2000). Refined structure of desmodium yellow mottle tymovirus at 2.7 Å resolution1. J. Mol. Biol. 301, 625–642. 10.1006/jmbi.2000.3983 10966774

[B58] Larson S. B. Lucas R. W. Greenwood A. McPherson A. (2005). The RNA of turnip yellow mosaic virus exhibits icosahedral order. Virology 334, 245–254. 10.1016/j.virol.2005.01.036 15780874

[B59] Legin R. Bass P. Etienne L. Fuchs M. (1993). Selection of mild virus strains of fanleaf degeneration by comparative field performance of infected grapevines. Vitis 32, 103–110.

[B60] Letunic I. Bork P. (2016). Interactive tree of life (iTOL) v3: an online tool for the display and annotation of phylogenetic and other trees. Nucleic Acids Res. 44, W242–W245. 10.1093/nar/gkw290 27095192 PMC4987883

[B61] Li P. Lin Y. Zhang H. Wang S. Qiu D. Guo L. (2016). Molecular characterization of a novel mycovirus of the family Tymoviridae isolated from the plant pathogenic fungus Fusarium graminearum. Virology 489, 86–94. 10.1016/j.virol,.2015.12.004 26744993

[B62] Londo J. P. Kovaleski A. P. Lillis J. A. (2018). Divergence in the transcriptional landscape between low temperature and freeze shock in cultivated grapevine (Vitis vinifera). Horticult. Res. 5:10. 10.1038/s41438-018-0020-7 29507734 PMC5830407

[B63] Martelli G. P. (2017). An overview on grapevine viruses, viroids, and the diseases they cause, in Grapevine Viruses: Molecular Biology, Diagnostics and Management, eds Meng B. Martelli G. P. Golino D. A. Fuchs M. (Cham: Springer International Publishing), 31–46. 10.1007/978-3-319-57706-7_2

[B64] Martelli P. G. Sabanadzovic S. N. Abou-Ghanem Sabanadzovic Edwards C. M. Dreher T. (2002). The family Tymoviridae. Arch. Virol. 147, 1837–1846. 10.1007/s007050200045 12209322

[B65] Massonnet M. Fasoli M. Tornielli G. B. Altieri M. Sandri M. Zuccolotto P.. (2017). Ripening transcriptomic program in red and white grapevine varieties correlates with berry skin anthocyanin accumulation. Plant Physiol. 174, 2376–2396. 10.1104/pp.17.00311 28652263 PMC5543946

[B66] McGovern P. E. (2003). Ancient Wine: The Search for the Origins of Viniculture. Princeton, NJ: Princeton University Press.

[B67] Monsion B. Incarbone M. Hleibieh K. Poignavent V. Ghannam A. Dunoyer P.. (2018). Efficient detection of long dsRNA *in vitro* and *in vivo* using the dsRNA binding domain from FHV B2 protein. Front. Plant Sci. 9:70. 10.3389/fpls.2018.00070 29449856 PMC5799278

[B68] Muller E. Ravel S. Agret C. Abrokwah F. Dzahini-Obiatey H. Galyuon I.. (2018). Next generation sequencing elucidates cacao badnavirus diversity and reveals the existence of more than ten viral species. Virus Res. 244, 235–251. 10.1016/j.virusres.2017.11.019 29169831

[B69] Nault L. R. Gingery R. E. Gordon D. T. (1980). Leafhopper transmission and host range of Maize Rayado Fino Virus. Phytopathology 70, 709–712. 10.1094/Phyto-70-709

[B70] Navarro B. Pantaleo V. Gisel A. Moxon S. Dalmay T. Bisztray G.. (2009). Deep sequencing of viroid-derived small RNAs from grapevine provides new insights on the role of RNA silencing in plant-viroid interaction. PLoS ONE 4:e7686. 10.1371/journal.pone.0007686 19890399 PMC2767511

[B71] Pearson W. R. Lipman D. J. (1988). Improved tools for biological sequence comparison. Proc. Natl. Acad. Sci. U. S. A. 85, 2444–2448. 10.1073/pnas.85.8.2444 3162770 PMC280013

[B72] Perry K. L. McLane H. Hyder M. Z. Dangl G. S. Thompson J. R. Fuchs M. F. (2016). Grapevine red blotch-associated virus is present in free-living *Vitis* spp. proximal to cultivated grapevines. Phytopathology 106, 663–670. 10.1094/PHYTO-01-16-0035-R 26960112

[B73] Peyret H. Lomonossoff G. P. (2013). The pEAQ vector series: the easy and quick way to produce recombinant proteins in plants. Plant Mol. Biol. 83, 51–58. 10.1007/s11103-013-0036-1 23479085

[B74] Poinar H. N. Schwarz C. Qi J. Shapiro B. R. MacPhee D. E. Schuster S. C.. (2006). Metagenomics to paleogenomics: large-scale sequencing of mammoth DNA. Science 311, 392–394. 10.1126/science.1123360 16368896

[B75] Poojari S. Alabi O. J. Fofanov V. Y. Naidu R. A. (2013). A leafhopper-transmissible DNA virus with novel evolutionary lineage in the family *Geminiviridae* implicated in grapevine redleaf disease by next-generation sequencing. PLoS ONE 8:e64194. 10.1371/journal.pone.0064194 23755117 PMC3673993

[B76] Qin J. Li R. Raes J. Arumugam M. Burgdorf K. S. Manichanh C. Wang J. (2010). A human gut microbial gene catalog established by metagenomic sequencing. Nature 464, 59–65. 10.1038/nature08821 20203603 PMC3779803

[B77] Roossinck M. J. (2011). The good viruses: viral mutualistic symbioses. Nat. Rev. Micro. 9, 99–108. 10.1038/nrmicro2491 21200397

[B78] Roossinck M. J. Saha P. Wiley G. B. Quan J. White J. D. Lai H.. (2010). Ecogenomics: using massively parallel pyrosequencing to understand virus ecology. Mol. Ecol. 19, 81–88. 10.1111/j.1365-294X.2009.04470.x 20331772

[B79] Saghai-Maroof M. A. Soliman K. M. Jorgensen R. A. Allard R. W. (1984). Ribosomal DNA spacer-length polymorphisms in barley: mendelian inheritance, chromosomal location, and population dynamics. Proc. Natl. Acad. Sci. U. S. A. 81, 8014–8018. 10.1073/pnas.81.24.8014 6096873 PMC392284

[B80] Saldarelli P. Giampetruzzi A. Maree H. J. Al Rwahnih M. (2017). High-throughput sequencing: advantages beyond virus identification, in Grapevine Viruses: Molecular Biology, Diagnostics and Management, eds Meng B. Martelli G. P. Golino D. A. Fuchs M. (Cham: Springer International Publishing), 625–642. 10.1007/978-3-319-57706-7_30

[B81] Silva J. M. F. Al Rwahnih M. Blawid R. Nagata T. Fajardo V. M. (2017). Discovery and molecular characterization of a novel enamovirus, Grapevine enamovirus-1. Virus Genes 53, 667–671. 10.1007/s11262-017-1470-y 28578531

[B82] Simmonds P. Adams M. J. Benko M. Breitbart M. Brister J. R. Carstens E. B.. (2017). Consensus statement: virus taxonomy in the age of metagenomics. Nat. Rev. Microbiol. 15, 161–168. 10.1038/nrmicro.2016.177 28134265

[B83] Stecher B. Denzler R. Maier L. Bernet F. Sanders M. J. Pickard D. J.. (2012). Gut inflammation can boost horizontal gene transfer between pathogenic and commensal Enterobacteriaceae. Proc. Natl. Acad. Sci. U. S. A. 109, 1269–1274. 10.1073/pnas.1113246109 22232693 PMC3268327

[B84] Suen G. Scott J. J. Aylward F. O. Adams S. M. Tringe S. G. Pinto-Tomás A. A.. (2010). An insect herbivore microbiome with high plant biomass-degrading capacity. PLoS Genet. 6:e1001129. 10.1371/journal.pgen.1001129 20885794 PMC2944797

[B85] Vargas-Asencio J. Wojciechowska K. Baskerville M. Gomez A. L. Perry K. L. Thompson J. R. (2017). The complete nucleotide sequence and genomic characterization of grapevine asteroid mosaic associated virus. Virus Res. 227, 82–87. 10.1016/j.virusres.2016.10.001 27720957

[B86] Vayssier-Taussat M. Albina E. Citti C. J. Cosson F. M. Jacques A. M. Candresse T. (2014). Shifting the paradigm from pathogens to pathobiome: new concepts in the light of meta-omics. Front. Cell. Infect.Microbiol. 4:29. 10.3389/fcimb.2014.00029 24634890 PMC3942874

[B87] Vigne E. Demangeat G. Komar V. Fuchs M. (2005). Characterization of a naturally occurring recombinant isolate of Grapevine fanleaf virus. Arch. Virol. 150, 2241–2255. 10.1007/s00705-005-0572-3 15968475

[B88] Vigne E. Garcia S. Komar V. Lemaire O. Hily J. M. (2004). Comparison of serological and molecular methods with high-throughput sequencing for the detection and quantification of Grapevine fanleaf virus (GFLV) in vineyard samples. Front. Microbiol. 35, 265–270. 10.1111/j.1365-2338.2005.00819.x PMC6262039 30524388

[B89] Vigne E. Komar V. Tannières M. Demangeat G. Duchêne E. Steyer D. (2015). Comparative pathogenic effects of distinct Grapevine fanleaf virus strains on Vitis vinifera cvs Gewurztraminer and Chardonnay, in 18th Congress of the International Council for the Study of Virus and Virus-Like Diseases of the Grapevine, ed ICVG, (Ankara), 236–237.

[B90] Wang L. Lv X. Zhai Y. Fu S. Wang D. Rayner S.. (2012). Genomic characterization of a novel virus of the family *Tymoviridae* isolated from mosquitoes. PLoS ONE 7:e39845. 10.1371/journal.pone.0039845 22848363 PMC3407206

[B91] White T. J. Bruns T. Lee S. Taylor J. (1990). Amplification and Direct Sequencing of Fungal Ribosomal RNA Genes for Phylogenetics, PCR Protocols. San Diego, CA: Academic Press, 315–322.

[B92] Yang J. Yan R. Roy A. Xu D. Poisson J. Zhang Y. (2015). The I-TASSER Suite: protein structure and function prediction. Nat. Meth. 12, 7–8. 10.1038/nmeth.3213 25549265 PMC4428668

[B93] Zenoni S. Ferrarini A. Giacomelli E. Xumerle L. Fasoli M. Malerba G.. (2010). Characterization of transcriptional complexity during berry development in *Vitis vinifera* using rna-seq. Plant Physiol. 152, 1787–1795. 10.1104/pp.109.149716 20118272 PMC2850006

[B94] Zhang Y. Singh K. Kaur R. Qiu W. (2011). Association of a novel DNA virus with the grapevine vein-clearing and vine decline syndrome. Phytopathology 101, 1081–1090. 10.1094/PHYTO-02-11-0034 21554183

[B95] Zhang Z. Qi S. Tang N. Zhang X. Chen S. Zhu P.. (2014). Discovery of replicating circular RNAs by RNA-seq and computational algorithms. PLoS Pathog. 10:e1004553. 10.1371/journal.ppat.1004553 25503469 PMC4263765

