# Supplementary material for: High-Throughput Sequencing and the Viromic Study of Grapevine Leaves: From the Detection of Grapevine-Infecting Viruses to the Description of a New Environmental Tymovirales Member
Source: Front Microbiol. 2018 Aug 29;9:1782. doi: 10.3389/fmicb.2018.01782 (PMC6123372; doi:10.3389/fmicb.2018.01782)
Supplement: Supplementary file 1 [file Presentation_1.PPTX]

## Slide 1
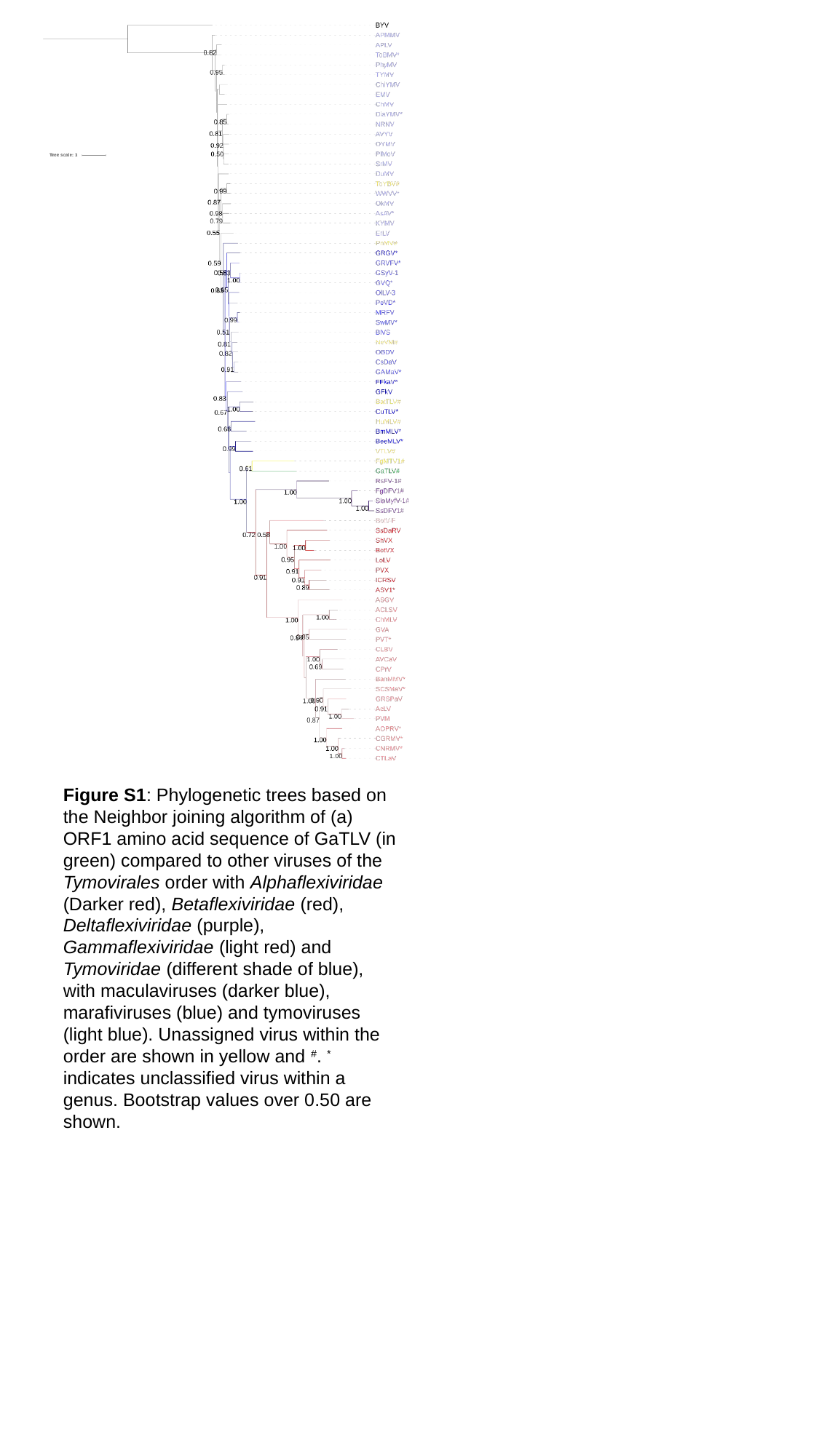

Figure S1: Phylogenetic trees based on the Neighbor joining algorithm of (a) ORF1 amino acid sequence of GaTLV (in green) compared to other viruses of the Tymovirales order with Alphaflexiviridae (Darker red), Betaflexiviridae (red), Deltaflexiviridae (purple), Gammaflexiviridae (light red) and Tymoviridae (different shade of blue), with maculaviruses (darker blue), marafiviruses (blue) and tymoviruses (light blue). Unassigned virus within the order are shown in yellow and #. * indicates unclassified virus within a genus. Bootstrap values over 0.50 are shown.

## Slide 2
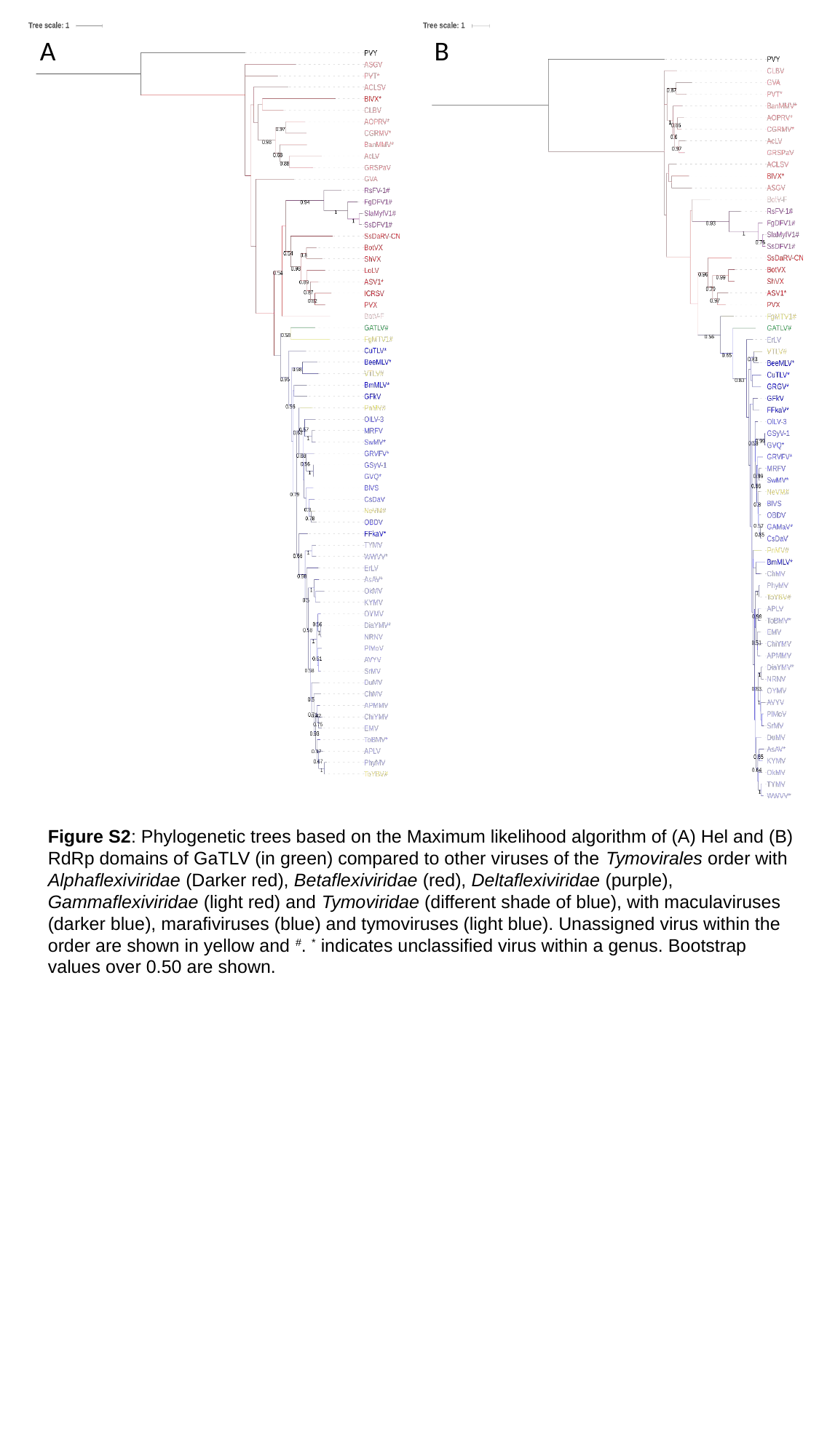

A
B
Figure S2: Phylogenetic trees based on the Maximum likelihood algorithm of (A) Hel and (B) RdRp domains of GaTLV (in green) compared to other viruses of the Tymovirales order with Alphaflexiviridae (Darker red), Betaflexiviridae (red), Deltaflexiviridae (purple), Gammaflexiviridae (light red) and Tymoviridae (different shade of blue), with maculaviruses (darker blue), marafiviruses (blue) and tymoviruses (light blue). Unassigned virus within the order are shown in yellow and #. * indicates unclassified virus within a genus. Bootstrap values over 0.50 are shown.

## Slide 3
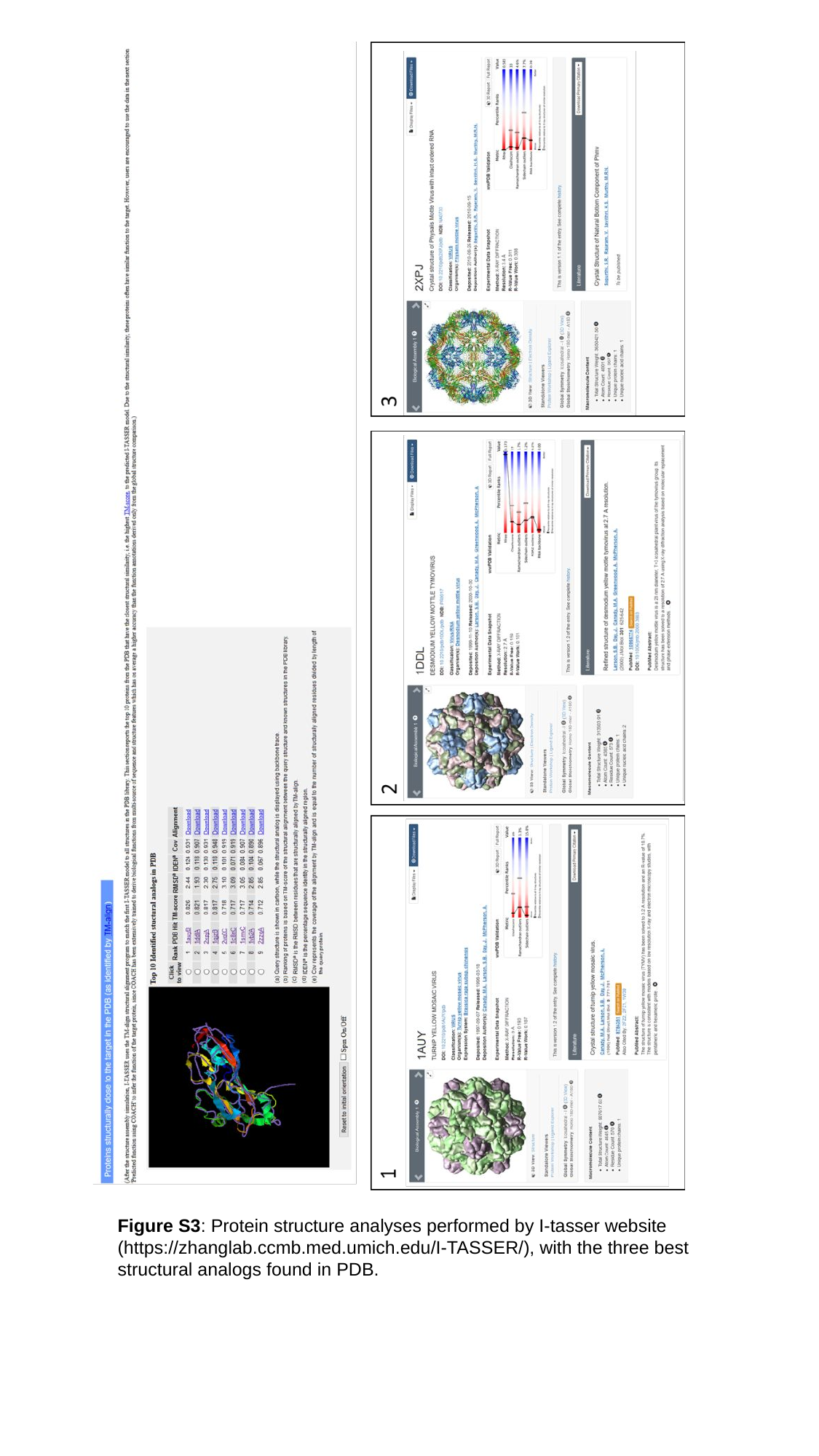

Figure S3: Protein structure analyses performed by I-tasser website (https://zhanglab.ccmb.med.umich.edu/I-TASSER/), with the three best structural analogs found in PDB.
