# Supplementary material for: High-Throughput Sequencing and the Viromic Study of Grapevine Leaves: From the Detection of Grapevine-Infecting Viruses to the Description of a New Environmental Tymovirales Member
Source: Front Microbiol. 2018 Aug 29;9:1782. doi: 10.3389/fmicb.2018.01782 (PMC6123372; doi:10.3389/fmicb.2018.01782)
Supplement: Supplementary file 2 [file Data_Sheet_1.docx]

**Table S1**: **List of viruses used for phylogenetic analyses, based on King et al., 2012**.


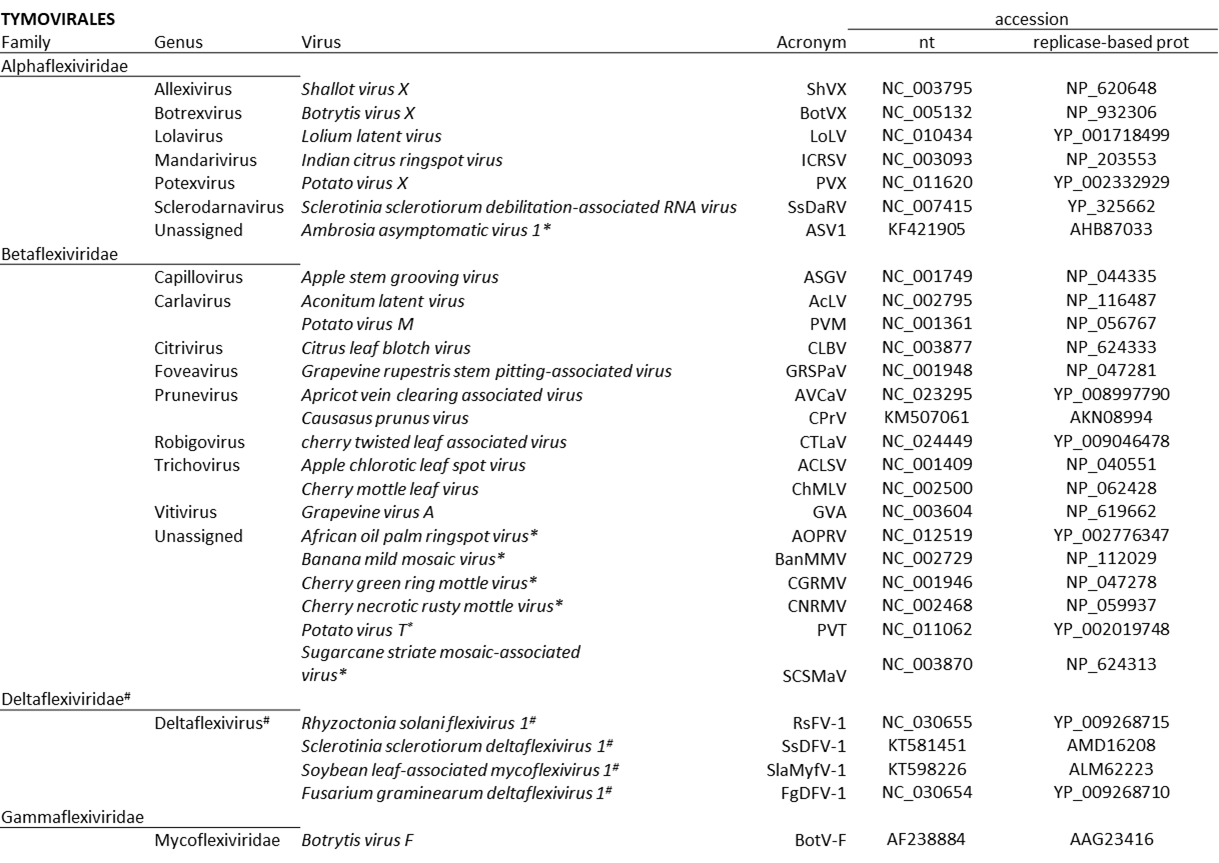


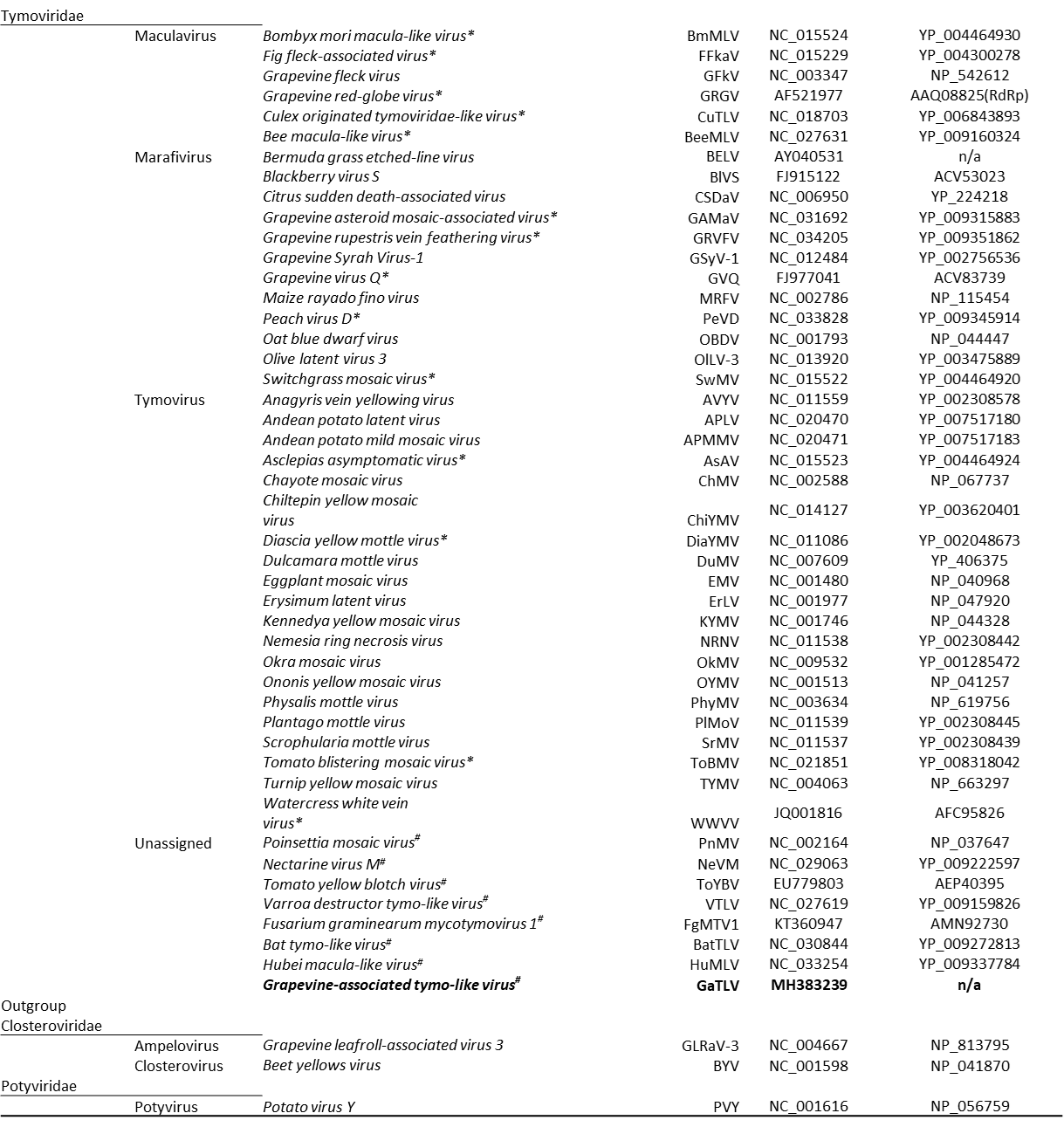


^#^ indicates unassigned virus within the order. ^*^ indicates unclassified virus within a genius. n/a: not available. N/A: Not Applicable.

**Table S2: Samples and viral sequences information.**


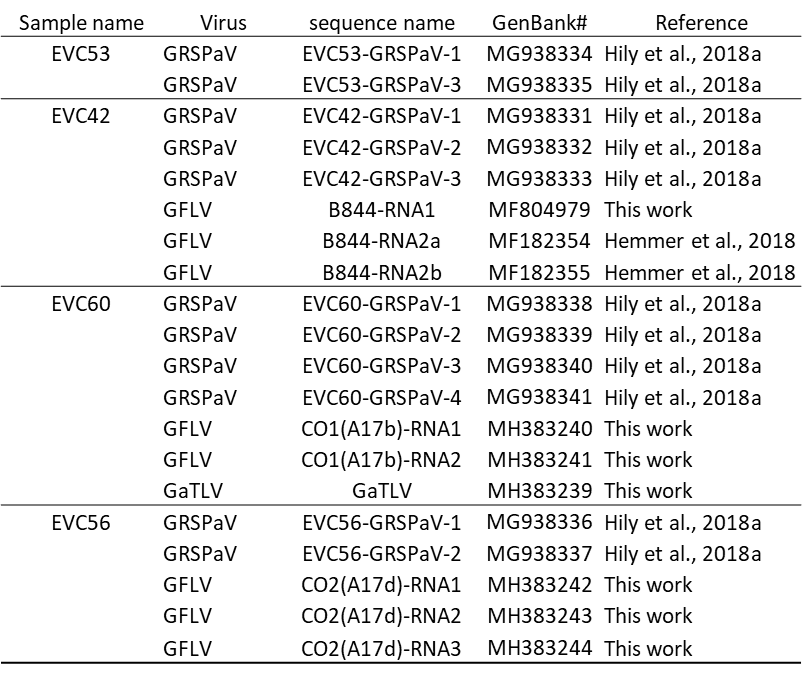


GRSPaV: grapevine rupestris stem pitting-associated virus, GFLV: grapevine fanleaf virus, GaTLV: grapevine-associated tymo-like virus.

**Table S3: Raw reads**


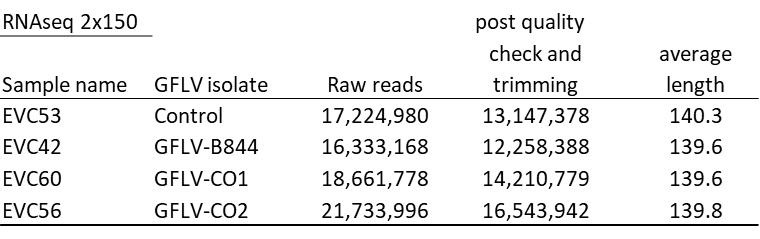


Total reads used in analyses post quality check and trimming, with average length.


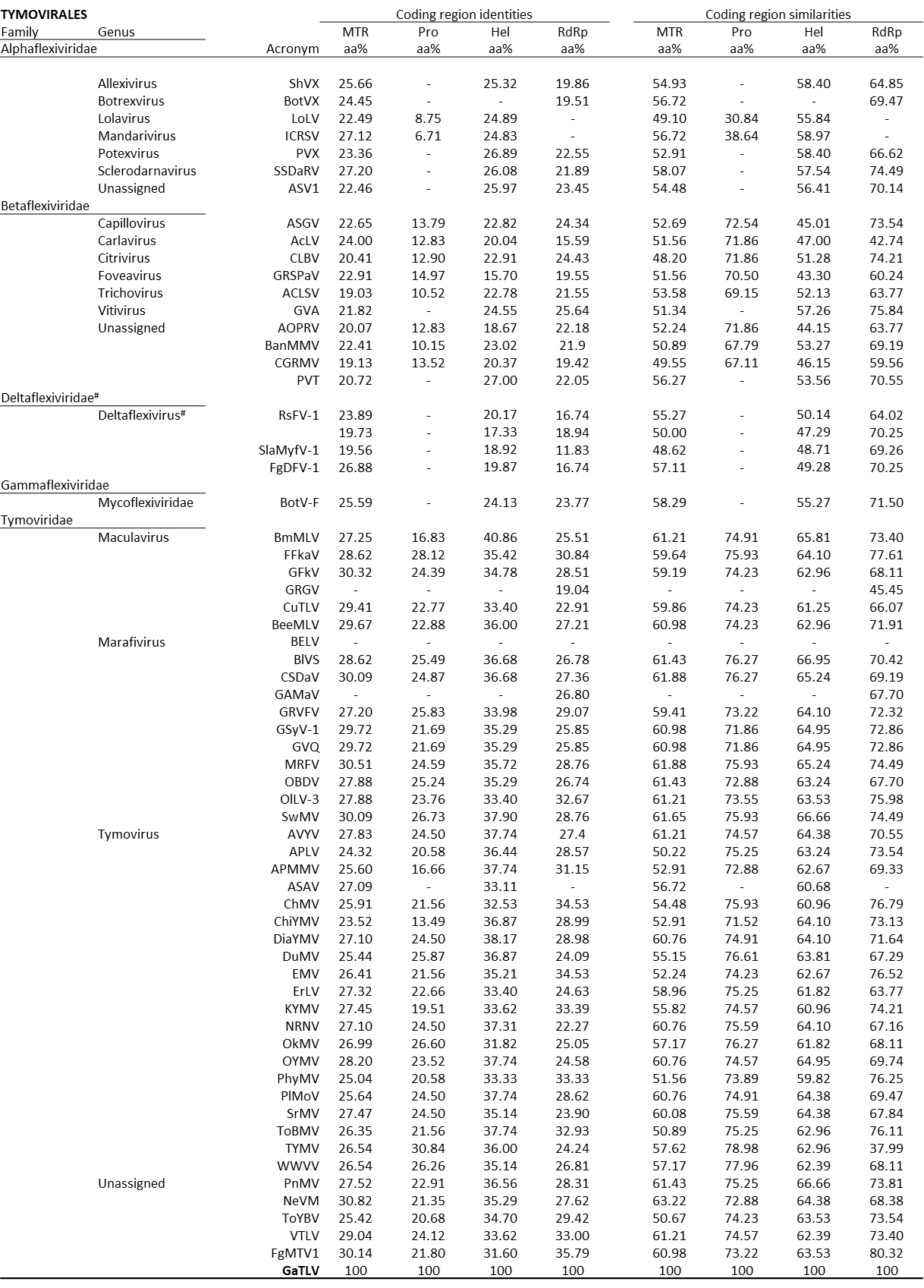
**Table S4**: **Amino acid sequence identities (%) and similarities (%) between GaTLV and typical viruses of the order *Tymovirales*.**

NT: Not Tested. - : no sequence available.
